# Characterization of the Role of Hexamer AGUAAA and Poly(A) Tail in Coronavirus Polyadenylation

**DOI:** 10.1371/journal.pone.0165077

**Published:** 2016-10-19

**Authors:** Yu-Hui Peng, Ching-Houng Lin, Chao-Nan Lin, Chen-Yu Lo, Tsung-Lin Tsai, Hung-Yi Wu

**Affiliations:** 1 Graduate Institute of Veterinary Pathobiology, College of Veterinary Medicine, National Chung Hsing University, Taichung, 40227, Taiwan ROC; 2 Department of Veterinary Medicine, National Pingtung University of Science and Technology, Neipu, Pingtung, 91201, Taiwan ROC; University of British Columbia, CANADA

## Abstract

Similar to eukaryotic mRNA, the positive-strand coronavirus genome of ~30 kilobases is 5’-capped and 3’-polyadenylated. It has been demonstrated that the length of the coronaviral poly(A) tail is not static but regulated during infection; however, little is known regarding the factors involved in coronaviral polyadenylation and its regulation. Here, we show that during infection, the level of coronavirus poly(A) tail lengthening depends on the initial length upon infection and that the minimum length to initiate lengthening may lie between 5 and 9 nucleotides. By mutagenesis analysis, it was found that (i) the hexamer AGUAAA and poly(A) tail are two important elements responsible for synthesis of the coronavirus poly(A) tail and may function in concert to accomplish polyadenylation and (ii) the function of the hexamer AGUAAA in coronaviral polyadenylation is position dependent. Based on these findings, we propose a process for how the coronaviral poly(A) tail is synthesized and undergoes variation. Our results provide the first genetic evidence to gain insight into coronaviral polyadenylation.

## Introduction

Posttranscriptional modifications occurring in the nucleus of eukaryotic cells include cleavage of the 3′ end of nascent mRNAs and the addition of a poly(A) tail [1–5]. The polyadenylation process involves two discrete phases [6]. In the first phase, synthesis of a short poly(A) tail of nearly 10 nucleotides (nts) depends on interaction between polyadenylation-related proteins and the polyadenylation signal (PAS) hexamer AAUAAA or its variant (AGUAAA, AUUAAA or UAUAAA) located 10–30 nts upstream of the poly(A) cleavage site [1, 7–13]. The rapid addition of a poly(A) tail of nearly 200 nts that occurs in the second phase requires the nearly 10 adenosine residues synthesized in the first phase. The synthesized poly(A) tail is important for the nuclear export of mature mRNAs and has been demonstrated to be involved in the control of mRNA stability and translation efficiency [14–17].

As opposed to mRNAs used only for translation, polyadenylation of viral RNA in RNA viruses may be involved in both translation and replication [16, 18]. RNA viruses have developed several mechanisms for synthesizing a poly(A) tail based on genetic features. It has been demonstrated that influenza virus utilizes a stretch of short U residues, instead of the hexamer AAUAAA, located at the 5’ terminus of the negative-strand genomic RNA as a signal for poly(A) synthesis by the viral RNA polymerase with a stuttering mechanism during positive-strand synthesis [19–21]. A similar mechanism is also used by paramyxoviruses to generate a poly(A) tail during transcription [22]. On the other hand, poliovirus uses homopolymeric stretch on negative-strand as template for the addition of poly(A) tail during positive-strand synthesis [23]. Moreover, the *cis*-acting element required for replication may also be used for polyadenylation in RNA viruses. For example, the hexamer AAUAAA in bamboo mosaic virus and a domain immediately upstream of the poly(A) tail in coxsackievirus B3 have been shown to function as *cis*-acting elements involved in both negative-strand RNA synthesis and polyadenylation [24, 25].

Bovine coronavirus (BCoV), a *betacoronavirus*, subfamily *Coronavirinae*, family *Coronaviridae* and order *Nidovirales*, is a 5’-capped and 3’-polyadenylated positive-strand RNA virus. Although the mechanism for coronaviral polyadenylation remains unknown, a stuttering mechanism based on the short poly(U) stretch found in the negative-strand genome has been postulated [26]. Moreover, a regulated poly(A) tail length during the coronaviruses life cycle has been suggested, whereby the viral poly(A) tail length is increased in the early stage of infection but gradually decreases after the peak tail length (~65 nts) in the later stage of infection in both cell culture and animals [16, 27]. Such regulated poly(A) tail length may function in translation regulation, as it has been experimentally demonstrated that a longer coronavirus poly(A) tail is associated with better translation efficiency [16]. However, the mechanism by which the coronaviral poly(A) tail is regulated remains unclear.

In the current study, we determined that both the poly(A) tail and hexamer AGUAAA are important elements responsible for the polyadenylation of coronavirus. The efficiency of poly(A) tail elongation during infection depends on the initial poly(A) tail length at the time of infection. Based on these findings, we propose a process for how the coronaviral poly(A) tail is synthesized and undergoes variation. The results presented here provide the first genetic evidence that will help in elucidating coronaviral polyadenylation.

## Materials and Methods

### Viruses and cell line

A plaque-purified Mebus strain of bovine coronavirus (BCoV) (from Dr. David A. Brian, University of Tennessee, USA) (GenBank accession no. U00735) at 3 × 10^7^ PFU/ml was used as a helper virus throughout the study. Human rectal tumor (HRT)-18 cells (from Dr. David A. Brian, University of Tennessee, USA) were maintained in DMEM supplemented with 10% fetal bovine serum and used for BCoV infection as described [28–30].

### Plasmid constructs

Construction of defective interfering (DI) RNA pW-25A (formerly called pBM25A) (S1A Fig, lower panel) in which the 288-nt 3’ UTR of BCoV-Mebus in BCoV DI RNA pDrep1 (S1A Fig, upper panel) was replaced with the 301-nt 3’ UTR and 25-nt poly(A) tail of mouse hepatitis virus (MHV)-A59 (GenBank accession no. NC_001846) has been described [16, 31, 32]. To construct pR-25A, an overlap PCR mutagenesis procedure was performed as previously described [31, 33, 34], but using oligonucleotides TGEV 7(−) and R(+) and pW-25A DNA in the first PCR, oligonucleotides R(−) and BM25A(+) and pW-25A DNA in the second PCR, and oligonucleotides TGEV 7(−) and BM25A(+) and the products of the first two reactions in the third PCR. The resulting PCR product was cloned into the TOPO XL vector (Invitrogen) and digested with *Spe*I and *Mlu*I. The digested fragment was then cloned into *Spe*I- and *Mlu*I-linearized pW-25A to generate the mutant pR-25A. Mutants of pM1-25A, pM2-25A, pM3-25A, pM4-25A, pM5-25A, pPAS-R-25A and pPAS-PAS-25A were similarly constructed, except for the corresponding oligonucleotides used in the first and second reactions, as described in S1 Table.

To generate the constructs pW-0A, pW-5A, pW-8A, pW-12A, pW-15A, pW-18, pW-20A, pW(C)-25A, pW-25U, pW-25C, pW-25G, pW-random and pW-polyCC, PCR was performed using pW-25A DNA as the template with the oligonucleotide TGEV 7(-) and the appropriate oligonucleotide that binds to the terminal sequence of the 3’ UTR, as described in S1 Table, to create DI RNA constructs with various patterns of sequences at their 3’ ends. Each PCR product was cloned into the TOPO-XL vector (Invitrogen) and digested with *Spe*I and *Mlu*I, and the digested fragments were cloned into *Spe*I- and *Mlu*I-linearized pW-25A to create the aforementioned constructs. For constructs pR-5A, pR-8A, pR-12A, pR-15A, pR-18, pR-20A, pR(C)-25A, pR-25U, pR-25C, and pR-25G, PCR was also performed using a similar method but with pR-25A DNA as the template. This strategy was also applied to generate mutants pM1-15A, pM2-15A, pM3-15A, pM4-15A, pM5-15A, pPAS-R-15A and pPAS-PAS-15A, but the DNA templates for these mutants were pM1-25A, pM2-25A, pM3-25A, pM4-25A, pM5-25A, pPAS-R-25A and pPAS-PAS-25A, respectively, and the oligonucleotides were TGEV 7(-) and BM15A(+).

### *In vitro* transcription and transfection

To synthesize transcripts *in vitro*, all DNA constructs (except W-0A, W-25U, W-25C, W25G, R-25U, R-25C and R-25G, which were linearized with *BsmB*I to accurately synthesize transcript with no poly(A) tail, or with only poly (U), poly(C) or poly(G) tail) were linearized with *Mlu*I. The linearized DNA was transcribed *in vitro* with the mMessage mMachine T7 transcription kit (Ambion) according to the manufacturer's instructions and passed through a Biospin 6 column (Bio-Rad), followed by transfection [35]. For transfection, HRT-18 cells in 35-mm dishes at ~80% confluency (~8 × 10^5^ cells/dish) were infected with BCoV at a multiplicity of infection of 5 PFU per cell. After 2 hours of infection, 3 μg of transcript was transfected into mock-infected or BCoV-infected HRT-18 cells using Lipofectine (Invitrogen) [31, 36].

### Preparation of RNA from infected cells

To prepare RNA for the identification of DI RNA poly(A) tail length, RNA was extracted with TRIzol (Invitrogen) at the indicated times after transfection of DI RNA constructs into BCoV-infected HRT-18 cells; the virus within the transfected cells is referred to as virus passage 0 (VP0) (S1B Fig). Supernatants from BCoV-infected and DI RNA transfected HRT-18 cells at 48 hours posttransfection (hpt) (VP0) were collected, and 500 μl was used to infect freshly confluent HRT-18 cells in a 35-mm dish (virus passage 1, VP1) (S1B Fig). RNA was extracted with TRIzol (Invitrogen) at the indicated time points.

### Determination of poly(A) tail length

Among the PCR-based methods for the determination of poly(A) tail length [37–41], a head-to-tail ligation method using tobacco acid pyrophosphatase (TAP) and RNA ligase followed by RT-PCR and sequencing was employed in this study (S1C Fig). This method has been previously used to identify the terminal features of histone mRNA [42] and influenza virus [43] as well as the poly(A) tail length of cellular mRNAs [44] and coronavirus RNAs [16, 27]. In brief, 10 μg of extracted total cellular RNA in 25 μl of water, 3 μl of 10X buffer and 10 U of (in 1 μl) TAP (Epicentre) were used to de-block the 5′ capped end of genomic RNA. Following decapping, RNA was phenol-chloroform-extracted, dissolved in 25 μl of water, heat-denatured at 95°C for 5 min and quick-cooled. Head-to-tail ligation was then performed by adding 3 μl of 10X ligase buffer and 2 U (in 2 μl) of T4 RNA ligase I (New England Biolabs); the mixture was incubated for 16 h at 16°C. The ligated RNA was phenol-chloroform-extracted and used for the RT reaction. SuperScript II reverse transcriptase (Invitrogen), which is able to transcribe poly(A) tails greater than 100 nts with fidelity [19, 45], was used for the RT reaction with oligonucleotide BCV29-54(+), which binds to nts 29–54 of leader sequence of the 5’ UTR of the BCoV positive strand, as previously described. A 5-μl aliquot of the resulting cDNA was used in a 50-μl PCR with AccuPrime Taq DNA polymerase (Invitrogen) and oligonucleotides BCV29-54(+) and MHV3UTR3(-), the latter of which binds to nts 99–122 counted from the poly(U) track on the MHVA59 negative strand. The resulting PCR product was subjected to sequencing to determine the poly(A) tail length. At least three independent experiments were carried out for determining the poly(A) tail length of DI RNA mutants.

### Northern blot analysis

Detection of the reporter-containing DI RNA was performed essentially as described previously [35, 36]. In brief, HRT-18 cells were seeded in 35-mm dishes at ~80% confluency (~8 × 10^5^ cells/dish). For RNA stability assay, 3 μg of DI RNA transcript was transfected into the HRT-18 cells. HRT-cells were incubated with RNase A (final concentration 0.5 mg/ml) for 15 min prior to extraction of cellular RNA. For a replication assay, after 2 h of infection with BCoV at a multiplicity of infection of 5 PFU per cell, 3 μg of DI RNA transcript was transfected into the BCoV-infected HRT-18 cells. Tthe supernatant was harvested at 48 hpt (VP0), and 500 μl was used to infect freshly confluent HRT-18 cells in a 35-mm dish (VP1). Total cellular RNA was extracted with TRIzol (Invitrogen) at 48 hpi of VP1, and 10 μg was electrophoresed through a formaldehyde-agarose gel. The RNA was transferred from the gel to a Nytran membrane by vacuum blotting, and the blot was probed with 5’-end ^32^P-labeled oligonucleotide TGEV8(+) for 16 h. The probed blot was washed and autoradiographed at -80°C for 24 h.

### Quantitation of DI RNA synthesis by qRT-PCR

To determine the replication efficiency of DI RNA, the cDNA prepared as described above from head-to-tail ligation RNA collected at 1 and 24 hpi of VP1 was used for real-time PCR amplification with TagMan^®^Universal PCR Master Mix (Applied Biosystems) using primers MHV3’UTR3(-) and BCV23-40(+). The real-time PCR amplification was performed according to the manufacturer’s recommendations in a LightCycler^®^ 480 instrument (Roche Applied Science).

## Results

### Determination of the minimum length of poly(A) tail required to initiate poly(A) tail lengthening of coronavirus defective interfering (DI) RNA

**Fig 1:**
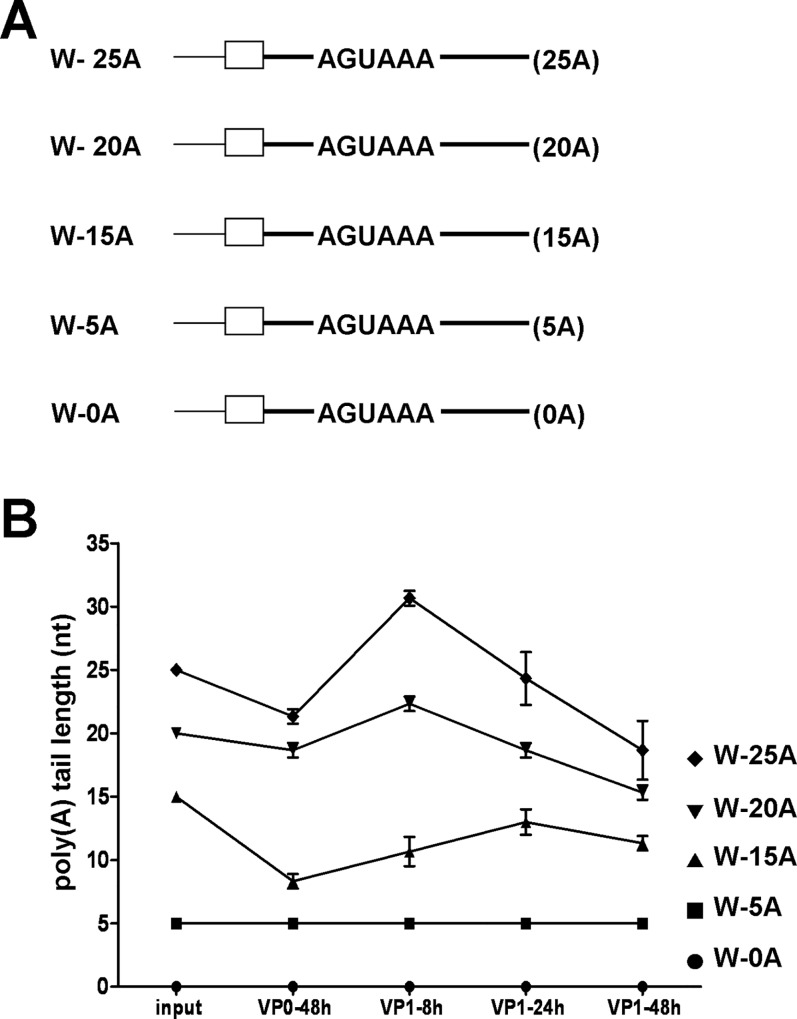
Determination of the minimum poly(A) tail length for the initiation of poly(A) tail lengthening. (A) DI RNA constructs with various poly(A) tail lengths. (B) Poly(A) tail lengths for DI RNA constructs at different times, as determined by sequencing the RT-PCR products shown in S2 Fig. Input: DI RNA transcript used for transfection, as shown in Fig 1A.

In a previous study, we demonstrated that the length of the coronaviral poly(A) tail on both viral RNAs and DI RNA is regulated during infection; that is, the poly(A) tail length is increased in the early stage of infection and then decreased after the peak tail length in the later stage of infection [16, 27]. To test whether the increase in coronaviral poly(A) tail length requires a minimum tail length in the initial viral RNA, as with the requirement of nearly 10 adenosine residues for the second phase of polyadenylation in eukaryotic mRNA, a series of bovine coronavirus (BCoV) DI RNAs with various poly(A) tail lengths were constructed and tested (Fig 1A). The 2.2-kb and helper virus-dependent BCoV DI RNA (S1A Fig, upper panel) is a naturally occurring DI RNA [36, 46], and it has been extensively exploited for analyzing the *cis*-acting elements required for replication in coronaviruses [16, 33, 35, 36, 47–49]. To differentiate the origin of the poly(A) tail between the helper virus BCoV genome and BCoV DI RNA, the latter was engineered to carry the mouse hepatitis virus (MHV) 3’ UTR (S1A Fig, lower panel) [16, 31, 32] with which an MHV-specific primer can be used for RT-PCR to determine the length of the DI RNA poly(A) tail [16, 27]. It should be noted that both BCoV and MHV-A59 belong to the genus *betacoronavirus* and that the replication efficiency of this MHV 3’ UTR-containing BCoV DI RNA is similar to that of wild-type BCoV DI RNA [32]. After transfection of DI RNA constructs into BCoV-infected HRT-18 cells, the virus within the transfected cells is referred to as virus passage 0 (VP0) (S1B Fig). Supernatants from BCoV-infected and DI RNA transfected HRT-18 cells at 48 hours posttransfection (hpt) (VP0) were collected, and 500 μl was used to infect freshly confluent HRT-18 cells in a 35-mm dish (virus passage 1, VP1) (S1B Fig). After RNA extraction and head-to-tail ligation (S1C Fig), RT-PCR products with the size of less than 200 bp (S2 Fig), which are expected to contain the sequence from 3’ UTR, poly(A) tail and 5’UTR of DI RNA (S1C Fig) [16], were detected and subjected to sequencing to determine the poly(A) tail length. The RT-PCR products with the size of less than 100 bp were also sequenced and were determined to be primer-dimer. Note that the amounts of DI RNAs in cells were various at different time points of infection and thus various cycles of PCR were applied to amplify sufficient amounts of products for sequencing. Accordingly, the intensity of RT-PCR product shown in S2 Fig may not represent the replication efficiency of the DI RNAs. As shown in Fig 1B, the tail length of DI RNAs W-0A and W-5A remained the same (0 and 5 nts, respectively) throughout infection. To ensure that the identified poly(A) tail is from the replicating DI RNA rather than input DI RNA, qRT-PCR was applied for the evaluation of the replication efficiency for DI RNAs W-0A and W-5A at different time points of VP1. As shown in the upper panels of S3A and S3B Fig, the intensity of RT-PCR product was increased with the time of infection in VP1, suggesting that these DI RNAs is able to replicate. The results were subsequently confirmed by qRT-PCR (S3A and S3B Fig, lower panels) and thus suggest that the detected poly(A) tails of W-0A and W-5A were not from input DI RNAs but from replicating DI RNAs. Conversely, the poly(A) tail length of DI RNA W-15A (with an initial poly(A) tail length of 15 nts) was decreased (8 nts) at 48 hpt (VP0), gradually increased (10 and 13 nts, respectively) at 8 and 24 hours postinfection (hpi) of VP1 and then decreased (11 nts) at 48 hpi of VP1. Although the poly(A) tail length of DI RNA W-20A (with an initial poly(A) tail length of 20 nts) was also shortened (19 nts) at 48 hpt (VP0), the tail length was increased (22 nts) at 8 hpi of VP1 and then gradually decreased (19 nts and 15 nts at 24 and 48 hpi of VP1, respectively). Similar results were obtained for DI RNA W-25A (with an initial poly(A) tail length of 25 nts), whereby the poly(A) tail length was also shorter (21 nts) than the initial length at 48 hpt (VP0) but increased to 31 nts at 8 hpi of VP1 and then gradually decreased to 24 nts and 19 nts at 24 and 48 hpi of VP1, respectively. Because it has been shown that the poly(A) tail length of BCoV DI RNA within infected cells at 48 hpt (VP0) is similar to that of packaged BCoV DI RNA in inoculum collected at the same time point [16], the poly(A) tail length of DI RNA in infected cells may represent that in inoculum at 48 hpt (VP0). Under this criterion and based on the results that (i) the coronaviral poly(A) tail length of DI RNA W-15A increased from 8 nts at 48 hpt (VP0) to 10 nts at 8 hpi of VP1 and (ii) the tail length of W-0A and W-5A remained the same during VP0 and VP1, we conclude that the minimum poly(A) tail length required to initiate tail lengthening of coronavirus DI RNA may lie between 5 and 9 nts during the natural infection of VP1, regardless of the length of the input DI RNA transcript. Moreover, the level of lengthening was found to be correlated to the initial length of the poly(A) tail; that is, DI RNA with a longer poly(A) tail (for example, W-25A) showed a better lengthening than that with a shorter poly(A) tail (for example, W-15A), as evidenced by the comparison of poly(A) tail lengths synthesized at 48h of VP0 and 8h of VP1 for these DI RNA constructs during infection (Fig 1B).

### Effect of hexamer AGUAAA and poly(A) tail length on the efficiency of coronaviral polyadenylation

**Fig 2:**
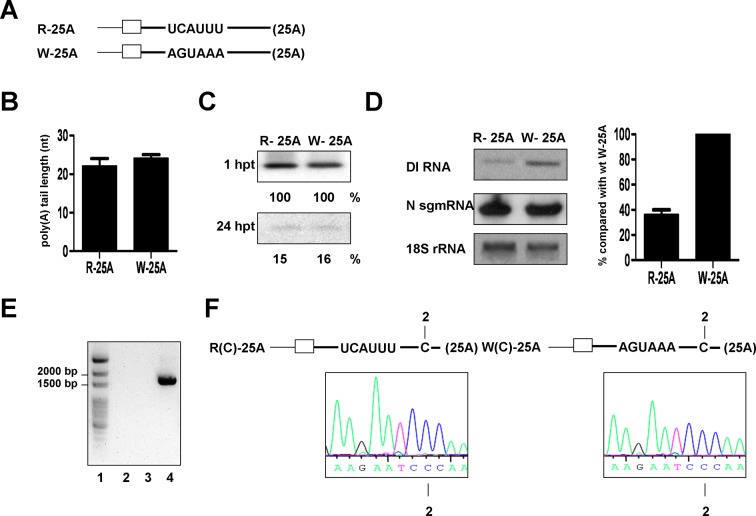
Effect of hexamer AGUAAA on polyadenylation of DI RNA with 25 nts of poly(A) tail. (A) DI RNA constructs with 25 nts of poly(A) tail in which hexamer AGUAAA was substituted with UCAUUU (R-25A) or maintained (W-25A). (B) lengths of R-25A and W-25A poly(A) tails at 24 hpi of VP1. (C) Stability of R-25A and W-25A in uninfected cells as measured by Northern blot assay. Transcripts were transfected and RNA was extracted at the times indicated. The amount of each DI RNA at 24 hpt was quantitated and compared with that at 1 hpt. (D) Left panel: the amounts of DI RNAs R-25A and W-25A at 24 hpi of VP1 as measured by Northern blot assay with BCoV N subgenomic mRNA (sgm RNA) and 18S rRNA as internal controls. Right panel: quantitation of the relative efficiency of replication between R-25A and W-25A. (E) Detection of potential recombination between DI RNA and BCoV genome at 24 hpi of VP1. The primer MHV3’UTR2(+), which anneals to the 3’ UTR of DI RNA and primer BM3(-),which anneals to the BCoV M protein gene were used for RT-PCR to identify the potential recombination between BCoV genome and R-25A (lane 2) or W-25A (lane 3). The recombinant DNA of 1,639 nts between DI RNA and BCoV genome shown in lane 4 was produced by overlap PCR and used as a size marker. Lane 1: ds DNA size markers. (F) Upper panel: DI RNA constructs R(C)-25A and W(C)-25A in which the nt at the position 2 upstream of poly(A) tail in DI RNAs R-25A and W-25A was mutated from A to C, respectively. Lower panel: sequence of the 3’ end of DI RNA at 24 hpi of VP1; the nt C at the position 2 upstream of poly(A) tail is given at the bottom of the sequence.

Interactions between consensus polyadenylation signal (PAS) hexamer AAUAAA or its variant (AGUAAA, AUUAAA or UAUAAA) located10–30 nts upstream of the poly(A) cleavage site [5, 10] and related proteins are integral aspects of eukaryotic mRNA polyadenylation [6, 50]. Although we in the previous study (Fig 1) have identified the minimum requirement to initiate lengthening of the coronaviral poly(A) tail during infection, little is known with regard to the detailed mechanism of how poly(A) tails are synthesized in coronaviruses. As with most eukaryotic mRNAs, both the coronavirus genome and subgenomic mRNAs are 3’ polyadenylated. Moreover, the PAS hexamer AGUAAA is also found in the 3’ UTR of genome and subgenomic mRNAs in BCoV and MHV-A59 between 37 and 42 nts upstream of the poly(A) site. To determine whether the hexamer AGUAAA, as with the eukaryotic PAS, serves as a *cis*-element involved in coronaviral polyadenylation, the hexamer in BCoV DI RNA W-25A was mutated from AGUAAA to UCAUUU; the resulting DI RNA was designated R-25A (Fig 2A). RNA was collected at 24 hpi of VP1, and the RT-PCR product was detected and subjected to sequencing analysis. As shown in Fig 2B, the lengths of the W-25A and R-25A poly(A) tails were 24 and 22 nts, respectively, suggesting that the hexamer AGUAAA only had a minor effect on coronavirus polyadenylation when the tail length of the input R-25A was 25 nts. However, under the similar RNA stability (Fig 2C), the replication efficiency of R-25A was impaired in comparison with that of W-25A, as determined by Northern blot analysis (36% vs 100%) (Fig 2D), suggesting that the replication efficiency may not be a major factor determining the poly(A) tail length. Besides, to exclude the possibility that the detected poly(A) tail is from the potential recombination between the DI RNA and BCoV genome, the primer MHV3’UTR2(+), which anneals to the 3’ UTR of DI RNA and primer BM3(-),which anneals to the BCoV M protein gene were used for RT-PCR to identify the potential recombinant [31, 32, 51]. However, no RT-PCR product was observed (Fig 2E, lanes 2–3), suggesting there is no potential DI RNA-BCoV genome recombinant synthesized during infection. Furthermore, because the last 21 nts of 3’ UTR between DI RNA and BCoV genome of helper virus are identical [52], it is also possible that the synthesized poly(A) tail may originate from genome of the helper virus BCoV via homologous recombination in this region either during negative- or positive-strand RNA synthesis. To test this possibility, the nt A at the position 2 upstream of poly(A) tail in DI RNAs R-25A and W-25A was mutated to C to create R(C)-25A and W(C)-25A as shown in Fig 2F, upper panel. As shown in Fig 2F, lower panel, the mutated nt C was still maintained at 24 hpi of VP1, suggesting that there is no homologous recombination between helper virus and DI RNA in this region and thus the detected poly(A) tail on DI RNAs R-25A and W-25A may not acquire from a potential DI RNA-BCoV genome recombination. It is noteworthy that the UCAUUU sequence in DI RNA mutant R-25A did not revert back to the wild-type AGUAAA at 24 hpi of VP1 (data not shown). Accordingly, the RNA stability (Fig 2C), replication efficiency (Fig 2D) and recombination between DI RNA and BCoV genome (Fig 2E and 2F) may not be the major factors in determining the poly(A) tail length on R-25A or W-25A.

**Fig 3:**
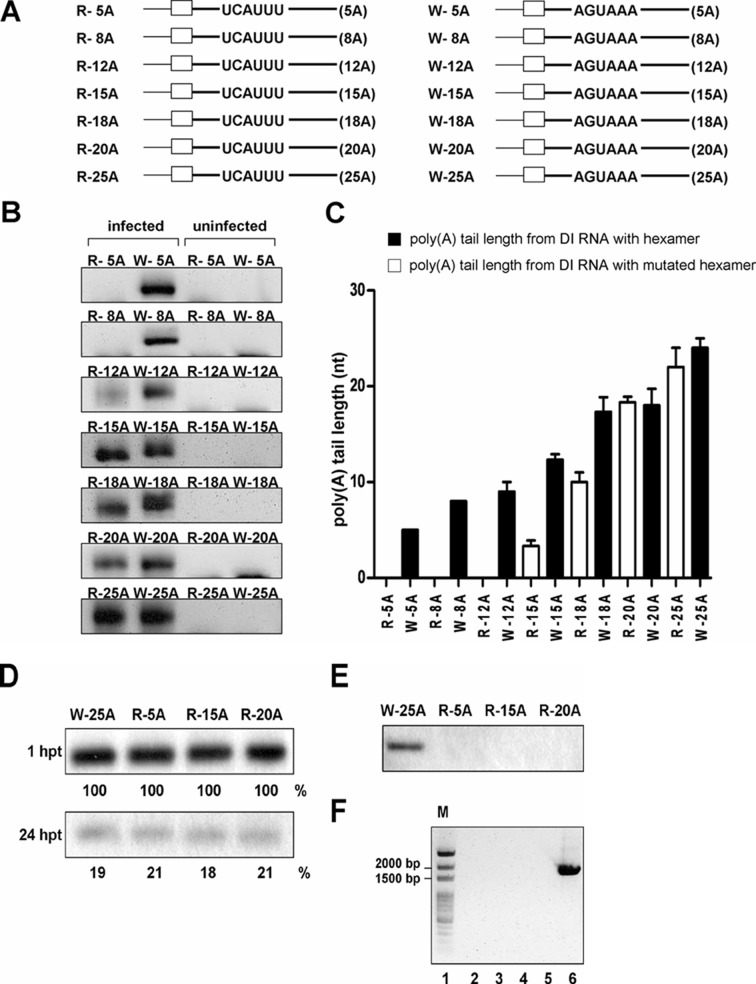
Effect of hexamer AGUAAA and poly(A) tail length on polyadenylation of DI RNA. (A) DI RNA constructs with various poly(A) tail lengths in which hexamer AGUAAA was substituted with UCAUUU (left panel) or maintained (right panel). (B) RT-PCR products synthesized using the method described in S1C Fig RNA samples were collected at 24 hpi of VP1 from cells treated with the supernatant from DI RNA-transfected BCoV-infected cells (left panel) or from DI RNA-transfected mock-infected cells (right panel). For uncropped gel images, see S3 Fig. (C) DI RNA poly(A) tail lengths, as determined by sequencing analysis of the RT-PCR products shown in Fig 3B. (D) Stability of DI RNA variants in uninfected cells as measured by Northern blot assay. The amount of each DI RNA at the times indicated was quantitated and compared with that at 1 hpt. (E) The amounts of DI RNA variants at 24 hpi of VP1 as measured by Northern blot assay. (F) Detection of potential recombination between DI RNAs W-25A (lane 2), R-5A (lane 3), R-15A (lane 4) or R-20A (lane 5) and BCoV genome. The recombinant DNA of 1,639 nts between DI RNA and BCoV genome shown in lane 6 was produced by overlap PCR and used as a size marker. Lane 1: ds DNA size markers.

Since the hexamer AGUAAA may not be required for coronaviral polyadenylation when the poly(A) tail on input DI RNA is 25 nts long, we hypothesize that the AUGAAA hexamer may be critical when the tail length is short. That is, it may be possible that both the hexamer AGUAAA and poly(A) tail may contribute to coronaviral polyadenylation through concerted action when the tail is at a certain length shorter than 25 nts on input DI RNA. This hypothesis is based on the results that (i) a poly(A) tail was not synthesized for W-0A (Fig 1B), even though this DI RNA construct contains a hexamer AGUAAA, and (ii) the level of poly(A) tail synthesis for hexamer AGUAAA-deficient R-25A was similar to that for hexamer AGUAAA-containing W-25A (Fig 2B). Therefore, to test our hypotheses and further elucidate the role of the AGUAAA hexamer in coronaviral polyadenylation, we created a series of DI RNA constructs in which the hexamer was substituted with UCAUUU and with various poly(A) tail lengths (Fig 3A, left panel) or the hexamer was intact but with various poly(A) tail lengths (Fig 3A, right panel). According to previous study, the W-65A (with 65 nts of poly(A) tail), which is structurally the same as W-25A (with 25 nts of poly(A) tail) except poly(A) tail length, is almost not detected at 1 and 2 hpi of VP1 but is steadily identified in the later stage (e.g. 24 hpi) of infection using head-to-tail ligation and RT-PCR [16] under the same amplification condition, suggesting the detected DI RNA in the later infection is newly synthesized; therefore, to ensure that the detected DI RNA is not from the input DI RNA which may be carried over by supernatant of VP0 but from the replicating DI RNA, total cellular RNA was collected at 24 hpi of VP1. As shown in Fig 3B (for uncropped gel images, see S4 Fig), RT-PCR products were observed for W-5A- or W-8A-transfected BCoV-infected cells at the same time point with poly(A) tail lengths of 5 and 8 nts, respectively (Fig 3C). Although RT-PCR products were observed for AGUAAA-deficient R-12A, no clear poly(A) tail was identified; instead, sequencing analysis revealed a mixed population at the 3’-terminal end. Nonetheless, an RT-PCR product was detected for W-12A, and after sequencing, the poly(A) tail length of W-12A was found to be 9 nts (Fig 3C). Interestingly, an RT-PCR product was also observed for AGUAAA-deficient R-15A, but subsequent sequencing revealed a poly(A) tail length of 3 nts (Fig 3C). On the other hand, the length of the W-15A poly(A) tail was determined to be 13 nts (Fig 3C). For R-18A, R-20A and R-25A, the poly(A) tail lengths were 10, 18, and 22 nts, respectively, whereas those for W-18A, W-20A and W-25A were 17, 19 and 24 nts, respectively (Fig 3C). Based on a comparison of synthesized poly(A) tail lengths between R-5A and W-5A, R-8A and W-8A, R-12A and W-12A, R-15A and W-15A, and R-18A and W-18A (Fig 3C), the poly(A) tail in AGUAAA-deficient DI RNA is shorter than that in AGUAAA-containing DI RNA. Accordingly, it was concluded that when the poly(A) tail length for the input DI RNA transcript is 18 nts or less, the hexamer AGUAAA is required for coronaviral polyadenylation. However, according to the results for R-20A, W-20A, R-25A and W-25A (Fig 3C), once the poly(A) tail length for the input DI RNA transcript reached 20 nts, the synthesized poly(A) tail length for the AGUAAA-deficient DI RNA was similar to that of AGUAAA-containing DI RNA. Thus, it was concluded that the hexamer AGUAAA is not required for polyadenylation when the tail length on input DI RNA transcript is 20 nts (for example, R-20A) or more (for example, R-25A). Because the poly(A) tail length of DI RNA became varied after transfection (Fig 1B) and the DI RNA poly(A) tail length at 48 hpt (VP0) in infected cells was similar to that of packaged DI RNA in inoculum [16], we also applied RT-PCR and sequencing to identify the poly(A) tail length of R-20A at 48 hpt (VP0). The length of poly(A) tail for R-20A was determined to be 18 nts at 48 hpt (VP0) (data not shown) and therefore it was also concluded that when the initial poly(A) tail length for coronavirus genome is 18 nts or more, coronaviral polyadenylation during natural infection is independent of hexamer AGUAAA. Taken together, the results suggest that (i) the poly(A) tail length plays an important role in the efficiency of coronaviral polyadenylation and (ii) the hexamer AGUAAA is also involved in coronaviral polyadenylation and may function in concert with the poly(A) tail to accomplish the subsequent polyadenylation when the initial length of poly(A) tail is shorter than 18 nts. This conclusion, therefore, supports our hypothesis.

To further determine whether, besides hexamer AGUAAA and poly(A) tail length, the DI RNA stability, replication efficiency and recombination are also factors affecting the synthesis of poly(A) tail on DI RNA, DI RNAs W-25A, R-5A, R-15A and R-20A with various length of synthesized poly(A) tail (24, 0, 3 and 18 nts, respectively) at 24 hpi of VP1 (Fig 3C) were selected and tested. As shown in Fig 3D, the stability of selected DI RNA variants is almost the same, suggesting the stability may not the main determinant affecting polyadenylation. For the factor of replication efficiency, DI RNA was not detectable by Northern blot assay from R-15A and R-20A, suggesting that the replication efficiency for both constructs is low (Fig 3E); however, the poly(A) tail length for both constructs was different (3 and 18 nts, respectively) (Fig 3C), also suggesting replication efficiency may not play a major role in poly(A) tail synthesis (see **Discussion** for more details). In addition, the unidentified poly(A) tail from R-5A may be attributed to the undetected RT-PCR product due to the poor replication efficiency. For the factor of recombination, no RT-PCR product was observed (Fig 3F, lanes 2–5) using the primers specifically binding to DI RNA and BCoV genome as described above (Fig 2E), suggesting that the detected poly(A) tail on the selected DI RNAs may not acquire from a potential DI RNA-BCoV genome recombinant. Furthermore, given that the poly(A) tail in DI RNAs originates from the genome or subgenome of the helper virus BCoV, the length in DI RNAs is expected to be longer than 40 nts because poly(A) tail in genome or subgenome of the helper virus BCoV at 8, 24 and 48 hpi is ~68, ~50 and ~40 nts, respectively [16]. However, the lengths of poly(A) tail in all DI RNAs used in the current study at these time points of infection are all shorter than 40 nts (the longest length is ~31 nts in W-25A at 8 hpi of VP1, Fig 1B). Therefore, consistent with the results shown in Fig 2 for R-25A and W-25A, DI RNA stability, replication efficiency and recombination may not the major factors in determining the synthesis of poly(A) tail on DI RNA.

### Synthesis of a poly(A) tail from poly(A) tail-lacking DI RNA

**Fig 4:**
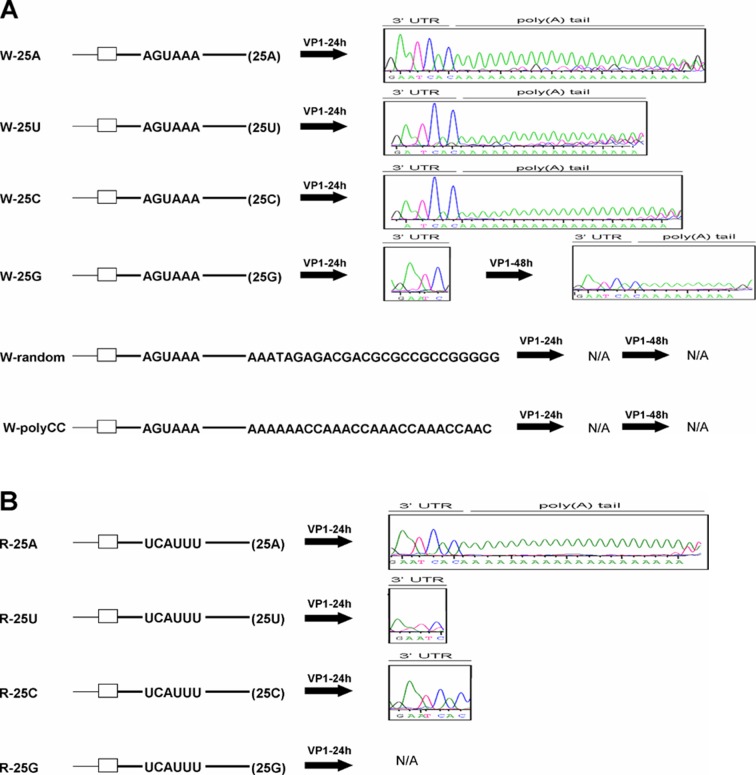
Synthesis of a poly(A) tail from poly(A) tail-lacking DI RNA. (A) Left panel: hexamer AGUAAA-containing DI RNA constructs with a poly(A) tail or other non-poly(A) sequences immediately downstream of the 3’UTR. Right panel: sequences of the 3’ end of DI RNA at the indicated times. (B) Left panel: hexamer AGUAAA-deficient DI RNA constructs with a poly(A) tail or other non-poly(A) sequences immediately downstream of the 3’UTR. Right panel: sequence of the 3’ end of DI RNA at 24 hpi of VP1. N/A: not available.

To test further the role of hexamer AGUAAA and poly(A) tail in coronaviral polyadenylation, 25 nts of the W-25A poly(A) tail was first replaced with the same length of a poly(U), poly(C) or poly(G) tail to create mutant W-25U, W-25C or W-25G, respectively (Fig 4A). Moreover, 25 nts of the W-25A poly(A) tail were also substituted with random sequences to generate mutants W-random and W-polyCC (Fig 4A). These mutants were then transfected into BCoV-infected cells to examine whether a poly(A) tail is synthesized and where on the DI RNA it is added if the tail is identified. As shown in Fig 4A, poly(A) tails with lengths of 18 and 21 nts were found on W-25U and W-25C. Moreover, the position where the tail was added was not at the 3’ terminus of the poly(U) or poly(C) tail but at the 3’ terminus of the 3’ UTR. For W-25G, a poly(A) tail was not found until 48 hpi of VP1, with an 8-nt tail also added to the 3’ terminus of the 3’ UTR. For W-random and W-polyCC, RT-PCR products were detected but no poly(A) tail or clear sequence of 3’ UTR was identified during VP1 infection. Although the mechanism remains to be elucidated, it was surprising that the poly(A) tail can be synthesized from DI RNA without poly(A) tail sequence such as W-25U, W-25C and W-25G. Regardless, since the poly(A) tail can be synthesized from DI RNA constructs W-25U, W-25C and W-25G, which contain a hexamer AGUAAA, they may be suitable candidates for further determining the requirement of the hexamer AGUAAA in poly(A) tail synthesis. That is, if the hexamer AGUAAA is mutated, lack of poly(A) tail synthesis for these hexamer AGUAAA-deficient constructs may reinforce the important role of the hexamer in coronaviral polyadenylation. To this end, the AGUAAA in W-25U, W-25C and W-25G was substituted with UCAUUU to create mutants R-25U, R-25C and R-25G. As shown in Fig 4B, the poly(A) tail was not found in R-25G because the RT-PCR product was not obtained. In this case, it was speculated that the poor replication efficiency may be the reason which leads to the result. However, when compared with W-25U and W-25C, a poly(A) tail was not synthesized for R-25U and R-25C at 24 and 48 hpi of VP1, further emphasizing the significant role of the AGUAAA hexamer in coronaviral polyadenylation.

### Dissection of hexamer AGUAAA by mutagenesis to further determine its role in coronavirus polyadenylation

**Fig 5:**
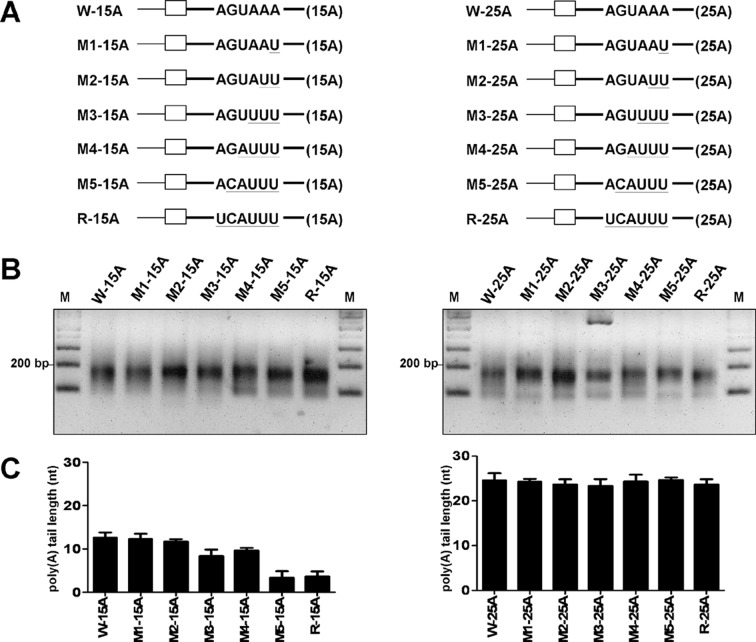
Dissection of hexamer AGUAAA to characterize its role in coronaviral polyadenylation. (A) DI RNA constructs with a series of mutations within hexamer AGUAAA and with a poly(A) tail of 15 (left panel) or 25 (right panel) nts in length. The mutated sequence is underlined. (B) RT-PCR products synthesized using the method described in Fig; RNA samples were harvested at 24 hpi of VP1. (C) The poly(A) tail length, as determined by sequencing the RT-PCR products shown in Fig 5B.

To further characterize the role of hexamer AGUAAA in coronaviral polyadenylation, the hexamer was dissected by mutagenesis (Fig 5A), and the effect on poly(A) tail synthesis was evaluated. RT-PCR products were detected for W-15A and other W-15A-derived mutants at 24 hpi of VP1, as displayed in Fig 5B, left panel. Sequencing analysis revealed that 1 (M1-15A) or 2 (M2-15A) substitutions from the 3’ end of the hexamer did not alter the efficiency of poly(A) tail synthesis, and the resulting length (13 nts) for these two mutants was the same as that for W-15A, which has an intact AGUAAA motif. However, decreased poly(A) tail synthesis efficiency was found for M3-15A (8 nts) and M4-15A (10 nts), with 3 and 4 substitutions, respectively, from the 3’ end of the hexamer. A severe impact on poly(A) tail synthesis occurred for M5-15A, in which 5 nts were substituted from the 3’ end of the hexamer, and the resulting poly(A) tail length was 3 nts, the same as that obtained for R-15A in which the entire hexamer was substituted. Therefore, the length of the poly(A) tail gradually decreases with the increased mutations within the hexamer. Based on the results that these DI RNAs were able to replicate (S5B Fig) and still retained the mutated hexamer sequence (S5C Fig), these results further support our argument that the AGUAAA hexamer functions as a *cis*-acting element in coronaviral polyadenylation. Additionally, different levels of substitutions within the hexamer AGUAAA were also performed in W-25A (Fig 5A, right panel). RT-PCR products were detected (Fig 5B, right panel), and the overall poly(A) tail lengths for the mutants M1-25A, M4-25A and M5-25A were not altered, whereas the lengths for M2-25A, M3-25A and R-25A are slightly decreased (Fig 5C, right panel), suggesting that the alternations in the poly(A) tail length do not occur in the DI RNAs with 25 nts of poly(A) tail and mutated AGUAAA. Taken together, these results further support our finding that the hexamer AGUAAA is an important *cis*-element in coronavirus polyadenylation.

### The function of hexamer AGUAAA in polyadenylation is position dependent

**Fig 6:**
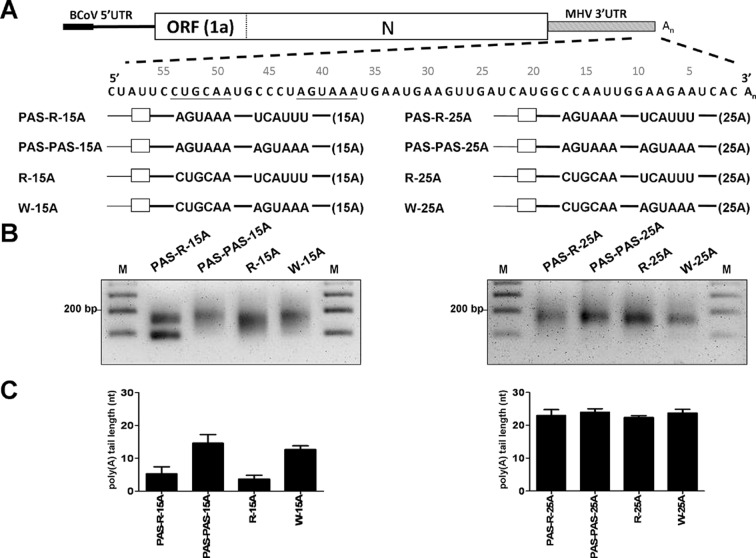
The function of hexamer AGUAAA in coronaviral polyadenylation is position dependent. (A) Upper panel: sequence within the 3’-terminal 60 nts of DI RNA. The number above the sequence indicates the position of nt counted from poly(A) tail. Lower panel: DI RNA constructs with a poly(A) tail of 15 (left panel) or 25 (right panel) nts in length, wherein the sequence (underlined) was mutated at position 37–42 (from hexamer AGUAAA to UCAUUU) and/or at position 49–54 (from CUGCAA to hexamer AGUAAA). (B) RT-PCR products synthesized from the method described in S1C Fig; RNA samples were collected at 24 hpi of VP1. (C) The poly(A) tail length, as determined by sequencing of the RT-PCR products shown in Fig 6B.

Based on the data presented here, this hexamer AGUAAA is involved in coronaviral polyadenylation. To determine whether the function of the hexamer AGUAAA in polyadenylation is position dependent, the sequence in W-15A between 37 and 42 nts upstream of the poly(A) tail site, where the original hexamer AGUAAA is located, was replaced with UCAUUU, and the sequence at 49 to 54 nts upstream of the poly(A) tail site was substituted with hexamer AGUAAA to create mutant PAS-R-15A (Fig 6A, left panel). In addition, mutant PAS-PAS-15A was also constructed in which the sequence between 49 and 54 nts upstream of the poly(A) tail site was replaced with the hexamer AGUAAA, but the original hexamer AGUAAA between 37 and 42 nts upstream of the poly(A) tail site was retained (Fig 6A, left panel). Wild-type DI RNA W-15A, mutant R-15A, in which the original hexamer AGUAAA was replaced with UCAUUU, and mutant PAS-PAS-15A were then used as controls to evaluate the position dependency of the hexamer AGUAAA in polyadenylation. Moreover, to ensure that the altered polyadenylation efficiency indeed results from sequence changes in the aforementioned positions of DI RNA with 15 nts of poly(A) tail, we also created constructs PAS-R-25A and PAS-PAS-25A, with poly(A) tail lengths of 25 nts (Fig 6A, right panel). We predicted that the sequence changes may not alter the polyadenylation efficiency for DI RNA with 25 nts of poly(A) tail according to the previous data shown in Fig 2. As shown in Fig 6B, left panel, RT-PCR products were observed for all constructs at 24 hpi of VP1, and the poly(A) tail lengths were determined to be 5, 15, 4 and 13 nts for PAS-R-15A, PAS-PAS-15A, R-15A and W-15A, respectively (Fig 6C, left panel). Note that the RT-PCR product for PAS-R-15A with the size less than 100 bp was sequenced and determined to be primer-dimer. These results suggest (i) the sequence substitution between 49 and 54 with hexamer AGUAAA in PAS-PAS-15A (the original hexamer AGUAAA was retained) did not affect the efficiency of polyadenylation when compared with the poly(A) tail length of W-15A (15 vs 13 nts) and (ii) the efficiency of poly(A) tail synthesis was still low (5 nts) for PAS-R-15A (the sequence between 49 and 54 was replaced with hexamer AGUAAA and the original hexamer AGUAAA was mutated). Note that these DI RNAs were able to replicate (S6B Fig) and the mutated sequences were still retained (S6C Fig). Consequently, since the position change of hexamer AGUAAA in PAS-R-15A did not restore the efficiency of poly(A) tail synthesis, it is concluded that the function of the hexamer AGUAAA in polyadenylation is position dependent. In addition, by RT-PCR (Fig 6B, right panel) and sequencing, the poly(A) tail length for constructs PAS-R-25A, PAS-PAS-PAS, R-25A and W-25A was identified to be similar (Fig 6C, right panel), suggesting that for DI RNA with poly(A) tail of 25 nts, substitution mutation between 49 and 54 nts or 37 and 42 nts upstream of the poly(A) site has only a minor or no effect on the efficiency of polyadenylation. Taken together, it is concluded that the function of the hexamer AGUAAA in coronaviral polyadenylation is position dependent.

### The hexamer AGUAAA or its variants are found among coronaviruses

The consensus PAS hexamer AAUAAA, located 10–30 nts upstream of the poly(A) cleavage site, is one of the *cis*-acting elements responsible for eukaryotic mRNA polyadenylation [1, 7, 8]. Variants, including AGUAAA, AUUAAA and UAUAAA, have also been identified as important for poly(A) tail synthesis in certain eukaryotic mRNA populations [9–11]. In the current study, we demonstrated that the hexamer AGUAAA, located between 37 and 42 nts upstream of the poly(A) site in BCoV, is involved in coronaviral polyadenylation. Moreover, the AGUAAA motif or its variants are also found in other coronaviruses, as summarized in Table 1. Among these hexamers, AAUAAA, AGUAAA and AUUAAA, which function in eukaryotic mRNA polyadenylation, are found in *betacoronavirus* C, *betacoronavirus* A and *deltacoronavirus*, respectively. Other hexamer variants, which differ from hexamer AAUAAA, AGUAAA or AUUAAA by one nucleotide, have also been identified in *alphacoronavirus*, *betacoronavirus* B, *betacoronavirus* D, and *gammacoronavirus*. Interestingly, although the positions of these hexamers in coronaviruses can be from 24 to 57 nts upstream of the poly(A) tail, the motifs are at similar positions within the same genus or lineage. However, it remains to be determined whether the hexamer has similar functions in coronaviruses other than BCoV.

**Table 1:** Postulated poly(A) signal in coronaviruses.

Virus^a^	Sequences	Position^b^	GenBank accession no.
**Alphacoronavirus**			
TGEV-Purdue	UGUAAA	39–34	DQ811788
FCoV	UGUAAA	39–34	NC_002306
HCoV-229E	AGUAAC	37–32	NC_002645
ScBtCoV-512	AGUAAC	36–31	NC_009657
PEDV	AGUAAC	37–32	NC_003436
MiBtCiV-HKU8	AGUAAU	38–33	NC_010438
**Betacoronavirus A**			
BCoV-Mebus	AGUAAA	42–37	U00735
HCoV-OC43	AGUAAA	42–37	NC_005147
PHEV-VW572	AGUAAA	42–37	NC_007732
ECoV	AGUAAA	42–37	NC_010327
MHV-A59	AGUAAA	42–37	NC_001846
MHV-JHM	AGUAAA	42–37	NC_006852
RbCoV-HKU14	AGUAAA	42–37	NC_017083
**Betacoronavirus B**			
SARS-CoV-Tor2	AAUUAA	57–52	NC_004718
BtSARS-CoV-Rp3	AAUUAA	57–52	DQ071615
BtSARS-CoV-HKU3	AAUUAA	57–52	DQ022305
**Betacoronavirus C**			
BtCoV-133/2005	AAUAAA	49–44	NC_008315
PiBTCoV-HKU5	AAUAAA	49–44	NC_009020
MERS-CoV	AAUAAA	49–44	NC_019843
**Betacoronavirus D**			
RoBtCoV-HKU9	AUUAUA	55–50	NC_009021
**Gammacoronavirus**			
IBV-Beaudette	AGUUAA	34–29	NC_001451
TCoV	AGUUAA	34–29	NC_010800
**Deltacoronavirus**			
PorCoV-HKU15	AUUAAA	29–24	NC_016990
SpCoV-HKU17	AUUAAA	29–24	NC_016882
MunCoV-HKU13	AUUAAA	29–24	NC_011550
MRCoV-HKU18	AUUAAA	29–24	NC_016993
BuCoV-HKU11	AUUAAA	29–24	FJ376619

a. TGEV-Purdue, porcine transmissible gastroenteritis virus Purdue strain; FCoV, feline infectious peritonitis virus; RhBtCoV-HKU2, *Rhinolophus* bat coronavirus HKU2; HCoV-NL63, human coronavirus NL63; HCoV-229E, human coronavirus 229E; SCBtCoV-512, *Scotophylus* bat coronavirus; PEDV, porcine epidemic diarrhea virus; MiBtCoV-HKU8, bat coronavirus HKU8; BCoV-Mebus, bovine coronavirus strain Mebus; HCoV-OC43, human coronavirus OC43; PHEV-VW572, porcine hemagglutinating encephalomyelitis virus; ECoV, equine coronavirus; MHV-A59, mouse hepatitis virus strain A59; MHV-JHM, mouse hepatitis virus strain JHM; RbCoV-HKU14, rabbit coronavirus HKU14; SARS-CoV-Tor2, SARS coronavirus isolateTor2; BtSARS-CoV-Rp3, bat SARS coronavirus Rp3; BtSARS-CoV-HKU3, bat SARS coronavirus HKU3; BtCoV-133/2005, bat coronavirus 133/2005; PiBTCoV-HKU5, *Pipistrellus* bat coronavirus HKU5; MERS-CoV, Middle East respiratory syndrome coronavirus or human betacoronavirus 2c EMC/2012; BtCoV-HKU9, bat coronavirus HKU9; IBV-Beaudette, avian infectious bronchitis virus strain Beaudette; TCoV, turkey coronavirus; PorCoV-HKU15, porcine coronavirus HKU15 strain HKU15-44; SpCoV-HKU17, sparrow coronavirus HKU17; MunCoV-HKU13, munia coronavirus HKU13-3514; MRCoV-HKU18, magpie-robin coronavirus HKU18; BuCoV-HKU11, bulbul coronavirus HKU11-934.

b. The number indicates the nucleotide position counted from ploy(A) tail.

## Discussion

In this article, we provide genetic evidence of how coronavirus lengthens its poly(A) tail during the infection cycle and how the hexamer AGUAAA and poly(A) tail act in concert to accomplish coronaviral polyadenylation. The characterization of two polyadenylation-related elements, AGUAAA and the poly(A) tail, will help in a better understanding of the mechanism of coronaviral polyadenylation.

### Factors involving in the regulation of coronaviral poly(A) tail length during coronavirus infection

In the previous study [16], it was found that the poly(A) tail length of W-65A (DI RNA with 65 nts of poly(A) tail) at 48 hpt (VP0) is 28 nts and was increased to be 62 nts at 8 hpi of VP1. The results led us to ask the question of what the the minimum poly(A) tail length required to initiate tail lengthening of coronavirus DI RNA is. In order to precisely identify the minimum requirement to initiate the lengthening of poly(A) tail, instead of constructing DI RNA with long ply(A) tail, for example, W-65A with 65 nts of poly(A) tail [16], several DI RNAs with a little variation in poly(A) tail length were constructed and therefore only a small variation in poly(A) tail length from these DI RNA constructs was expected in the process of infection as shown in Fig 1. In terms of the identification of the minimum length for the initiation of poly(A) lengthening, the small increase in the poly(A) tail length of the same DI RNA construct (for example, 2 nts increase in the poly(A) tail length of W-15A between 48 hpt (VP0) and 8 hpi of VP1) is therefore critical for the interpretation of the results although the level of length change is not dramatic. Regardless, the results shown in Fig 1 along with the previous findings from W-65A [16] collectively reinforce the critical nature of the initial poly(A) tail length in the elongation of coronaviral poly(A) tail in the early stage of infection. Interestingly, after 8 h of infection in VP1, the poly(A) tail length on DI RNAs W-25A and W-20A decreased from 31 and 22 nts to 24 and 19 nts, respectively, whereas that of DI RNA W-15A increased from 11 to 13 nts. We speculate that DI RNA with a short enough poly(A) tail may not be efficiently detected by deadenylases [53, 54], thus avoiding efficient tail degradation mediated by the deadenylation mechanism. After initial lengthening of the poly(A) tail, it is also feasible that the polyadenylation may occur again based on the previous lengthening poly(A) tail possibly by viral polymerase or cytoplasmic poly(A) polymerase during infection. On the other hand, in terms of the shortening of coronavial poly(A) tail, it is still not clear at this point what mechanism is responsible for the coronaviral deadenylation. The regulation of tail length in coronaviruses, therefore, may be complicated because it occurs in a dynamic process and in addition to poly(A) tail length, other factors such as deadenylase and viral polymerase or cytoplasmic poly(A) polymerase may be collectively involved in the decision of the eventual length of coronavirus poly(A) tails at a given time during infection through unidentified mechanisms.

### Contribution of hexamer AGUAAA for virus survival

In the present study, we demonstrated that both the poly(A) tail and hexamer AGUAAA are involved in coronaviral polyadenylation and the two elements appear to be able to functionally compensate for each other when one is missing or modified, as exemplified by DI RNA mutants R-25A and W-25U. Based on present results, we propose that both the hexamer AGUAAA and poly(A) tail contribute to virus survival under diverse environments. For example, a poly(A) tail length shorter than 20 nts was consistently found for coronaviral RNA collected from (i) BCoV-infected HRT-18 cells at 2 hpi, (ii) W-25A-transfected BCoV-infected HRT-18 cells at 72 hpi of VP1 and (iii) mouse brain at 5 days postinfection with MHV-A59 (S7 Fig). Moreover, in persistent infection, a MHV-A59 poly(A) tail length of less than 20 nts was also frequently found (S7 Fig); therefore, when such persistent-infection coronavirus infects fresh cells, the AGUAAA hexamer is also required in concert with the poly(A) tail for efficient polyadenylation. Thus, the hexamer AGUAAA along with the poly(A) tail may be required to restore efficient polyadenylation for subsequent translation and replication, contributing to virus survival under these conditions.

### The importance of the minor changes in poly(A) tail length for gene expression of coronavirus

Unlike most mammalian mRNAs with ~250 nts of poly(A) tail, positive-strand RNA viruses such as piconaviruses, have heterogeneous lengths of natural poly(A) tail ranging from about 10 to 120 nts long [55–57]. Functional analysis reveals that 12 nts of poly(A) tail on positive-strand genome of poliovirus is the minimum length to initiate negative-strand synthesis of genome and that increasing the poly(A) tail length from 12 to 13 nts results in about a ten-fold efficiency in negative-strand synthesis [58], suggesting that minor changes in viral poly(A) tail length exert significant effect on viral replication. Similar results have also been obtained in sindbis virus during negative-strand synthesis in which increasing the poly(A) tail length from 10 to 15 nts leads to a nearly nine-fold increase in negative-strand RNA synthesis [59]. The size of poly(A) tail in BCoV varies at different time points of infection and its distribution during infection is from about 13 to 68 nts (S7 Fig and [16]). As with aforementioned positive-strand RNA viruses, small alternations in poly(A) tail size from 5 to 10 nts and from 25 to 45 nts in coronavirus dramatically increase the efficiency of viral replication [18] and translation [16], respectively, further demonstrating the small changes in the poly(A) tail length play an important role in the biological function of coronavirus. Therefore, the lengthening of poly(A) tail, for example, in W-15A from 8 nts at 48 hpt of VP0 to 13 nts at 24 hpi of VP1 and W-25A from 21 nts at 48 hpt of VP0 to 31 nts at 8 hpi of VP1 (Fig 1B) may increase the efficiency of gene expression. Since the minor changes have an important effect on viral replication and translation, we speculate that for coronavirus with short poly(A) tail length ranging between ~13 to ~68 nts (in comparison with mammalian mRNA with ~250 nts of poly(A) tail) the minor changes in the poly(A) tail length may be sensitive and thus critical in the regulation of gene expression during infection.

### Evaluation of the effect of replication efficiency on polyadenylation of DI RNA

In the present study, we found that the replication efficiency of DI RNA mutants R-25A (36%) (Fig 2) and W-25C (<1%, S8 Fig) was much lower than that of W-25A (100%). However, the synthesized poly(A) tail lengths of these two mutants was similar to that of W-25A, suggesting that the replication efficiency may not be a major factor affecting coronaviral polyadenylation. For DI RNA with shorter poly(A) tail and AGUAAA mutation, for example, R-15A, its replication efficiency was similar to that of W-15A at the stage of 24 hpi of VP1,and RT-PCR products were detected for sequencing (S5A and S5B Fig); however, the synthesized poly(A) tail length (3 and 13 nts for R-15A and W-15A, respectively) was different (Fig 3C), also suggesting that the replication efficiency may not be a major factor in the poly(A) tail synthesis. Accordingly, this may be applied to account for the results shown in Fig 3E. The replication efficiency for both R-15A and R-20A was similar because they were not detected by Northern blot assay (Fig 3E); however, both were detected by RT-PCR and sequencing results showed that the poly(A) tail length for both constructs was different (3 and 18 nts, respectively) (Fig 3C), also suggesting replication efficiency may not play a major role in poly(A) tail synthesis. For R-5A in Fig 3E, the unidentified poly(A) tail from R-5A may be attributed to the undetected RT-PCR product due to the poor replication efficiency. The aforementioned arguments may also account for the results shown in Fig 4. For W-25U and W-25C, the replication efficiency for both DI RNAs is low because they were not detected by Northern blot assay (S8A Fig) when compared with that for W-25A. Subsequent study showed that the replication efficiency was similar between W-25U, W-25C, R-25U and R-25C as determined by qRT-PCR (S8B Fig); however, poly(A) tail was synthesized from W-25U and W-25C but not from R-25U and R-25C (Fig 4B), suggesting replication efficiency may not be the main determinant in the poly(A) tail synthesis. For R-25G, similar to R-5A, the unidentified poly(A) tail may be attributed to the undetected RT-PCR product. Taken together, we speculate that the replication efficiency may not be a major factor affecting coronaviral polyadenylation and that replication and polyadenylation in coronaviruses are separate processes but proceed by a similar theme during viral RNA synthesis.

### Role of hexamer AGUAAA in coronavirus replication

It has been shown that with reverse genetic approaches the deletion of nts 30–170 upstream of poly(A) tail do not affect the growth function in tissue culture for MHV [60, 61]. It is reasonable to speculate that the hexamer AGUAAA, located between 37 and 42 nts upstream of the poly(A) site, may not play a role in the replication. However, in the current study, the results suggest that the replication efficiency is decreased in DI RNA R-25A with mutated hexamer AGUAAA in comparison with that in W-25A with intact AGUAAA (36% vs 100%). We speculate that the system (reverse genetics with a full-length cDNA vs DI RNA) and mutagenesis (deletion of entire region between nts 30–170 vs replacement of hexamer within the undeleted region of nts 30–170) employed for the analysis may lead to the discrepancy of the results. Nevertheless such discrepancy does not affect the role of hexamer AGUAAA in the coronaviral polyadenylation concluded in this study because the polyadenylation appears not to be influenced by replication efficiency as evidenced by the results of DI RNA constructs discussed above.

### Proposed mechanism for coronaviral polyadenylation

**Fig 7:**
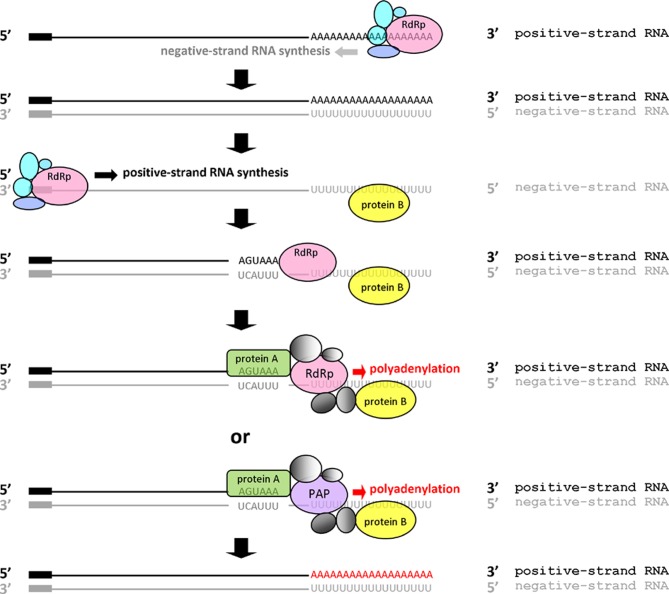
The proposed mechanism for polyadenylation in coronaviruses. Coronavirus replication complex utilizes positive-strand viral RNA as a template to synthesize negative-strand viral RNA with a poly(U) tract. The nascent negative-strand RNA in turn serves as a template for synthesizing positive-strand RNA. After hexamer AGUAAA on the positive-strand RNA is copied, binding of related proteins to the motif on the positive strand and that of polyadenylation-related proteins to the poly(U) tract on the negative strand along with coronavirus RNA-dependent RNA polymerase (RdRp) or cytoplasmic poly(A) polymerase (PAP) form a polyadenylation complex for poly(A) tail synthesis.

Based on the evidence shown in this study and in others, we propose a model for coronaviral polyadenylation (Fig 7). First, coronavirus polymerase utilizes positive-strand viral RNA as a template to synthesize negative-strand viral RNA in which the length of the poly(U) tract is similar to that of the poly(A) tail at the same time point after infection [16]. Subsequently, the negative-strand viral RNA serves a template for synthesizing the positive-strand viral RNA. During positive-strand viral RNA synthesis, once the nascent hexamer AGUAAA is copied, cytoplasmic polyadenylation-associated factors such as CPSF and other accessory factors may interact with the hexamer on the positive strand. This hexamer-protein complex may interact with (an)other complex(es) formed by interactions between proteins and the poly(U) tract on the negative strand to generate a stable RNA-protein complex that directs the viral RdRp to synthesize the poly(A) tail. Alternatively, the RNA-protein complex formed may recruit cytoplasmic poly(A) polymerase instead of viral RdRp for poly(A) tail synthesis [62]. This model emphasizes the important role of the AGUAAA hexamer on the nascent positive-strand viral RNA as well as the 5’ terminal sequence (i.e., poly(U) tract) on the negative-strand viral RNA, with which polyadenylation-associated proteins interact.

Accordingly, under the argument of constitution of a stable complex for efficient coronavirus polyadenylation, this model may explain (i) why a poly(A) tail was synthesized from poly(A)-deficient DI RNA constructs W-25U, W-25C, and W-25G, rather than poly(A)-deficient DI RNA W-0A, even though they all contain a hexamer AGUAAA; and (ii) why a poly(A) tail was found for R-15A and R-18A, but not R-25U, R25C and R25G, despite the fact that they all lack the hexamer AGUAAA. In the cases of W-25U, W-25C and W-25G, which contain the hexamer AGUAAA but lack a poly(A) tail, it is speculated that in spite of different affinity the binding of proteins (e.g., PABP [63, 64]) to the 5’ end of poly(A), poly(G) or poly(C) on the W-25U, W-25C or W-25G negative strand, respectively, and then to proteins binding to the AGUAAA motif on the nascent positive-strand RNA to form a protein complex is a key step in coronaviral polyadenylation. In contrast, in the case of W-0A, the aforementioned protein complex is not formed because there is no such RNA element at the 5’ end of negative-strand W-0A for protein binding and thus no polyadenylation occurs. Regarding constructs R-15A, R-18A, R-25U, R25C and R25G, in which the hexamer AGUAAA was mutated, we reason that if polyadenylation-related protein binding to RNA elements at the 5’ end of negative-strand viral RNA is not sufficient to form a stable RNA-protein complex, assistance from interactions between the AGUAAA motif and proteins may be required for polyadenylation. Accordingly, although proteins were able to bind to poly(A), poly(G) or poly(C) on the negative strand of R-25U, R-25C or R-25G, respectively, polyadenylation was unable to occur without the help of the hexamer AGUAAA to form a stable RNA-protein complex. Furthermore, under the same argument of constitution of a stable complex for efficient coronavirus polyadenylation, this model may be extended to explain why different levels of hexamer AGUAAA mutation (Fig 5) and the alternation of hexamer AGUAAA position (Fig 6) decreased the efficiency of polyadenylation on DI RNA.

In conclusion, we for the first time determined the viral RNA elements involved in coronaviral polyadenylation. Future works may elaborate on the identification of the cellular and viral proteins participating in the synthesis of the coronaviral poly(A) tail as well as the detailed mechanism of coronaviral polyadenylation and its regulation.

## Supporting Information

S1 Fig Diagram of BCoV DI RNA and the strategy for determining coronaviral poly(A) tail length. (A) The structure of BCoV DI RNA and its composition relative to the BCoV genome are illustrated in the upper panel. The engineered BCoV DI RNA with the MHV 3’ UTR (W-25A) used in this study is shown in the lower panel. To differentiate the origin of the poly(A) tail between the helper virus BCoV genome and BCoV DI RNA, the latter was engineered to carry the mouse hepatitis virus (MHV) 3’ UTR with which an MHV-specific primer can be used for RT-PCR to determine the length of the DI RNA poly(A) tail. (B) Schematic illustration of the experiment showing the transfection of BCoV-infected HRT-18 cells with DI RNA and the passage of the resulting BCoV progeny. (C) Method for determining the coronaviral poly(A) tail length. The decapped and head-to-tail ligated BCoV DI RNA was used as the template for RT-PCR with primer 1 (for RT) and primer 2 followed by sequencing.
(TIF)

S2 Fig RT-PCR product synthesized from the DI RNA constructs in Fig 1A using the method described in S1C Fig. (TIF)

S3 Fig Determination of replication for W-0A and W-5A. (A) and (B) Upper panel: detection of DI RNAs W-0A and W-5A at the different time points of VP1 by RT-PCR. (A) and (B) Lower panel: quantitation of the synthesis of DI RNAs W-0A and W-5A at the different time points of VP1 by qRT-PCR.
(TIF)

S4 Fig Uncropped images for RT-PCR gels as shown in Fig 3B. (TIF)

S5 Fig Replication analysis for DI RNAs with mutation within hexamer AGUAAA. (A) Detection of DI RNA synthesis at 1 and 24 hpi of VP1 by RT-PCR. (B) The relative levels of DI RNA synthesis at 1 and 24 hpi of VP1 as quantitated by qRT-PCR. (C) Sequence analysis of DI RNA at 24 hpi of VP1. The sequence within the hexamer AGUAAA of individual DI RNA different from that of DI RNA W-15A is underlined.
(TIF)

S6 Fig Replication analysis for DI RNAs PAS-R-15A, PAS-PAS-15A, R-15A and W-15A. (A) Detection of DI RNA synthesis at 1 and 24 hpi of VP1 by RT-PCR. (B) The relative levels of DI RNA synthesis at 1 and 24 hpi of VP1 as quantitated by qRT-PCR. (C) Sequence analysis of DI RNA at 24 hpi of VP1. The sequence of individual DI RNA different from that of DI RNA W-15A is underlined.
(TIF)

S7 Fig Poly(A) tail lengths under different environments. (A) BCoV-infected HRT-18 cells at 2 hpi (13 nts). (B) W-25A-transfected BCoV-infected HRT-18 cells at 72 hpi of VP1 (13 nts). (C) Mouse brain at 5 days postinfection with MHV-A59 (17 nts) (shown in negative strand). (D) Delayed brain tumor (DBT) cells with MHV persistent infection of 97 days (15 nts) (shown in negative strand).
(TIF)

S8 Fig Determination of efficiency of DI RNA synthesis. (A) Upper panel: synthesis of W-25A, W-25U, W-25C and W-25G at 48 hpi of VP1 as detected by Northern blot analysis. Lower panel: the relative levels of RNA synthesis. (B) Quantitation of the synthesis of DI RNAs W-25U, W-25C, R-25U and R-25C at 24 hpi of VP1 by qRT-PCR.
(TIF)

S1 Table Oligonucleotides used for this study. (TIF)

## References

[1] Wickens M, Stephenson P. Role of the conserved AAUAAA sequence: four AAUAAA point mutants prevent messenger RNA 3' end formation. Science. 1984;226(4678):1045–51. Epub 1984/11/30. . 6208611 10.1126/science.6208611

[2] Montell C, Fisher EF, Caruthers MH, Berk AJ. Inhibition of RNA cleavage but not polyadenylation by a point mutation in mRNA 3' consensus sequence AAUAAA. Nature. 1983;305(5935):600–5. Epub 1983/10/13. . 6194440 10.1038/305600a0

[3] Humphrey T, Christofori G, Lucijanic V, Keller W. Cleavage and polyadenylation of messenger RNA precursors in vitro occurs within large and specific 3' processing complexes. EMBO J. 1987;6(13):4159–68. Epub 1987/12/20. 3127203 10.1002/j.1460-2075.1987.tb02762.x PMC553899

[4] Chen F, MacDonald CC, Wilusz J. Cleavage site determinants in the mammalian polyadenylation signal. Nucleic Acids Res. 1995;23(14):2614–20. Epub 1995/07/25. 5s0253 [pii]. 7651822 10.1093/nar/23.14.2614 PMC307082

[5] Proudfoot NJ. Ending the message: poly(A) signals then and now. Genes Dev. 2011;25(17):1770–82. Epub 2011/09/08. 10.1101/gad.17268411 25/17/1770 [pii]. 21896654 PMC3175714

[6] Sheets MD, Wickens M. Two phases in the addition of a poly(A) tail. Genes Dev. 1989;3(9):1401–12. Epub 1989/09/01. . 2575065 10.1101/gad.3.9.1401

[7] Sheets MD, Ogg SC, Wickens MP. Point mutations in AAUAAA and the poly (A) addition site: effects on the accuracy and efficiency of cleavage and polyadenylation in vitro. Nucleic Acids Res. 1990;18(19):5799–805. Epub 1990/10/11. 2170946 10.1093/nar/18.19.5799 PMC332317

[8] Zarkower D, Stephenson P, Sheets M, Wickens M. The AAUAAA sequence is required both for cleavage and for polyadenylation of simian virus 40 pre-mRNA in vitro. Mol Cell Biol. 1986;6(7):2317–23. Epub 1986/07/01. 3023928 10.1128/mcb.6.7.2317-2323.1986 PMC367784

[9] Beaudoing E, Freier S, Wyatt JR, Claverie JM, Gautheret D. Patterns of variant polyadenylation signal usage in human genes. Genome Res. 2000;10(7):1001–10. Epub 2000/07/19. 10899149 10.1101/gr.10.7.1001 PMC310884

[10] Tian B, Hu J, Zhang H, Lutz CS. A large-scale analysis of mRNA polyadenylation of human and mouse genes. Nucleic Acids Res. 2005;33(1):201–12. Epub 2005/01/14. 33/1/201 [pii] 10.1093/nar/gki158 15647503 PMC546146

[11] Kamasawa M, Horiuchi J. Identification and characterization of polyadenylation signal (PAS) variants in human genomic sequences based on modified EST clustering. In Silico Biol. 2008;8(3–4):347–61. Epub 2008/11/27. 2008080028 [pii]. . 19032167

[12] Keller W, Bienroth S, Lang KM, Christofori G. Cleavage and polyadenylation factor CPF specifically interacts with the pre-mRNA 3' processing signal AAUAAA. EMBO J. 1991;10(13):4241–9. Epub 1991/12/01. 1756731 10.1002/j.1460-2075.1991.tb05002.x PMC453176

[13] Bienroth S, Keller W, Wahle E. Assembly of a processive messenger RNA polyadenylation complex. EMBO J. 1993;12(2):585–94. Epub 1993/02/01. 8440247 10.1002/j.1460-2075.1993.tb05690.x PMC413241

[14] Fuke H, Ohno M. Role of poly (A) tail as an identity element for mRNA nuclear export. Nucleic Acids Res. 2008;36(3):1037–49. Epub 2007/12/22. gkm1120 [pii] 10.1093/nar/gkm1120 18096623 PMC2241894

[15] Preiss T, Muckenthaler M, Hentze MW. Poly(A)-tail-promoted translation in yeast: implications for translational control. RNA. 1998;4(11):1321–31. Epub 1998/11/14. 9814754 10.1017/s1355838298980669 PMC1369706

[16] Wu HY, Ke TY, Liao WY, Chang NY. Regulation of Coronaviral Poly(A) Tail Length during Infection. PLoS One. 2013;8(7):e70548 Epub 2013/08/08. 10.1371/journal.pone.0070548 PONE-D-13-03509 [pii]. 23923003 PMC3726627

[17] Weiss EA, Gilmartin GM, Nevins JR. Poly(A) site efficiency reflects the stability of complex formation involving the downstream element. EMBO J. 1991;10(1):215–9. Epub 1991/01/01. 1671216 10.1002/j.1460-2075.1991.tb07938.x PMC452632

[18] Spagnolo JF, Hogue BG. Host protein interactions with the 3' end of bovine coronavirus RNA and the requirement of the poly(A) tail for coronavirus defective genome replication. J Virol. 2000;74(11):5053–65. Epub 2000/05/09. 10799579 10.1128/jvi.74.11.5053-5065.2000 PMC110857

[19] Poon LL, Fodor E, Brownlee GG. Polyuridylated mRNA synthesized by a recombinant influenza virus is defective in nuclear export. J Virol. 2000;74(1):418–27. Epub 1999/12/10. 10590131 10.1128/jvi.74.1.418-427.2000 PMC111553

[20] Poon LL, Pritlove DC, Fodor E, Brownlee GG. Direct evidence that the poly(A) tail of influenza A virus mRNA is synthesized by reiterative copying of a U track in the virion RNA template. J Virol. 1999;73(4):3473–6. Epub 1999/03/12. 10074205 10.1128/jvi.73.4.3473-3476.1999 PMC104115

[21] Zheng H, Lee HA, Palese P, Garcia-Sastre A. Influenza A virus RNA polymerase has the ability to stutter at the polyadenylation site of a viral RNA template during RNA replication. J Virol. 1999;73(6):5240–3. Epub 1999/05/11. 10233995 10.1128/jvi.73.6.5240-5243.1999 PMC112577

[22] Hausmann S, Garcin D, Delenda C, Kolakofsky D. The versatility of paramyxovirus RNA polymerase stuttering. J Virol. 1999;73(7):5568–76. Epub 1999/06/11. 10364305 10.1128/jvi.73.7.5568-5576.1999 PMC112614

[23] Steil BP, Kempf BJ, Barton DJ. Poly(A) at the 3' end of positive-strand RNA and VPg-linked poly(U) at the 5' end of negative-strand RNA are reciprocal templates during replication of poliovirus RNA. J Virol. 2010;84(6):2843–58. Epub 2010/01/15. JVI.02620-08 [pii] 10.1128/JVI.02620-08 20071574 PMC2826026

[24] Chen IH, Chou WJ, Lee PY, Hsu YH, Tsai CH. The AAUAAA motif of bamboo mosaic virus RNA is involved in minus-strand RNA synthesis and plus-strand RNA polyadenylation. J Virol. 2005;79(23):14555–61. Epub 2005/11/12. 79/23/14555 [pii] 10.1128/JVI.79.23.14555-14561.2005 16282455 PMC1287560

[25] van Ooij MJ, Polacek C, Glaudemans DH, Kuijpers J, van Kuppeveld FJ, Andino R, et al Polyadenylation of genomic RNA and initiation of antigenomic RNA in a positive-strand RNA virus are controlled by the same cis-element. Nucleic Acids Res. 2006;34(10):2953–65. Epub 2006/06/02. 34/10/2953 [pii] 10.1093/nar/gkl349 16738134 PMC1474053

[26] Hofmann MA, Brian DA. The 5' end of coronavirus minus-strand RNAs contains a short poly(U) tract. J Virol. 1991;65(11):6331–3. Epub 1991/11/01. 1920635 10.1128/jvi.65.11.6331-6333.1991 PMC250348

[27] Shien JH, Su YD, Wu HY. Regulation of coronaviral poly(A) tail length during infection is not coronavirus species- or host cell-specific. Virus Genes. 2014;49(3):383–92. Epub 2014/07/19. 10.1007/s11262-014-1103-7 . 25034371 PMC7089208

[28] King B, Brian DA. Bovine coronavirus structural proteins. J Virol. 1982;42(2):700–7. Epub 1982/05/01. 7086972 10.1128/jvi.42.2.700-707.1982 PMC256895

[29] Lapps W, Hogue BG, Brian DA. Sequence analysis of the bovine coronavirus nucleocapsid and matrix protein genes. Virology. 1987;157(1):47–57. Epub 1987/03/01. 0042-6822(87)90312-6 [pii]. . 3029965 10.1016/0042-6822(87)90312-6 PMC7130558

[30] Tompkins WA, Watrach AM, Schmale JD, Schultz RM, Harris JA. Cultural and antigenic properties of newly established cell strains derived from adenocarcinomas of the human colon and rectum. J Natl Cancer Inst. 1974;52(4):1101–10. Epub 1974/04/01. . 4826581 10.1093/jnci/52.4.1101

[31] Liao WY, Ke TY, Wu HY. The 3'-terminal 55 nucleotides of bovine coronavirus defective interfering RNA harbor cis-acting elements required for both negative- and positive-strand RNA synthesis. PLoS One. 2014;9(5):e98422 Epub 2014/05/24. 10.1371/journal.pone.0098422 PONE-D-14-04439 [pii]. 24852421 PMC4031142

[32] Wu HY, Brian DA. Subgenomic messenger RNA amplification in coronaviruses. Proc Natl Acad Sci U S A. 2010;107(27):12257–62. Epub 2010/06/22. 1000378107 [pii] 10.1073/pnas.1000378107 20562343 PMC2901459

[33] Ozdarendeli A, Ku S, Rochat S, Williams GD, Senanayake SD, Brian DA. Downstream sequences influence the choice between a naturally occurring noncanonical and closely positioned upstream canonical heptameric fusion motif during bovine coronavirus subgenomic mRNA synthesis. J Virol. 2001;75(16):7362–74. Epub 2001/07/20. 10.1128/JVI.75.16.7362-7374.2001 11462008 PMC114971

[34] Yeh PY, Wu HY. Identification of cis-acting elements on positive-strand subgenomic mRNA required for the synthesis of negative-strand counterpart in bovine coronavirus. Viruses. 2014;6(8):2938–59. Epub 2014/08/01. 10.3390/v6082938 v6082938 [pii]. 25080125 PMC4147681

[35] Wu HY, Ozdarendeli A, Brian DA. Bovine coronavirus 5'-proximal genomic acceptor hotspot for discontinuous transcription is 65 nucleotides wide. J Virol. 2006;80(5):2183–93. Epub 2006/02/14. 80/5/2183 [pii] 10.1128/JVI.80.5.2183-2193.2006 16474126 PMC1395388

[36] Chang RY, Hofmann MA, Sethna PB, Brian DA. A cis-acting function for the coronavirus leader in defective interfering RNA replication. J Virol. 1994;68(12):8223–31. Epub 1994/12/01. 7966615 10.1128/jvi.68.12.8223-8231.1994 PMC237289

[37] Salles FJ, Richards WG, Strickland S. Assaying the polyadenylation state of mRNAs. Methods. 1999;17(1):38–45. Epub 1999/03/17. S1046-2023(98)90705-8 [pii] 10.1006/meth.1998.0705 . 10075881

[38] Janicke A, Vancuylenberg J, Boag PR, Traven A, Beilharz TH. ePAT: a simple method to tag adenylated RNA to measure poly(A)-tail length and other 3' RACE applications. RNA. 2012;18(6):1289–95. Epub 2012/05/01. 10.1261/rna.031898.111 rna.031898.111 [pii]. 22543866 PMC3358650

[39] Patil DP, Bakthavachalu B, Schoenberg DR. Poly(A) polymerase-based poly(A) length assay. Methods Mol Biol. 2014;1125:13–23. Epub 2014/03/05. 10.1007/978-1-62703-971-0_2 24590776 PMC3951053

[40] Meijer HA, Bushell M, Hill K, Gant TW, Willis AE, Jones P, et al A novel method for poly(A) fractionation reveals a large population of mRNAs with a short poly(A) tail in mammalian cells. Nucleic Acids Res. 2007;35(19):e132 Epub 2007/10/16. gkm830 [pii] 10.1093/nar/gkm830 17933768 PMC2095794

[41] Beilharz TH, Preiss T. Transcriptome-wide measurement of mRNA polyadenylation state. Methods. 2009;48(3):294–300. Epub 2009/02/24. 10.1016/j.ymeth.2009.02.003 S1046-2023(09)00025-5 [pii]. . 19233282

[42] Mullen TE, Marzluff WF. Degradation of histone mRNA requires oligouridylation followed by decapping and simultaneous degradation of the mRNA both 5' to 3' and 3' to 5'. Genes Dev. 2008;22(1):50–65. Epub 2008/01/04. 10.1101/gad.1622708 22/1/50 [pii]. 18172165 PMC2151014

[43] Szymkowiak C, Kwan WS, Su Q, Toner TJ, Shaw AR, Youil R. Rapid method for the characterization of 3' and 5' UTRs of influenza viruses. J Virol Methods. 2003;107(1):15–20. . 12445933 10.1016/s0166-0934(02)00184-2

[44] Kojima S, Sher-Chen EL, Green CB. Circadian control of mRNA polyadenylation dynamics regulates rhythmic protein expression. Genes Dev. 2012;26(24):2724–36. Epub 2012/12/20. 10.1101/gad.208306.112 26/24/2724 [pii]. 23249735 PMC3533077

[45] Komine Y, Kwong L, Anguera MC, Schuster G, Stern DB. Polyadenylation of three classes of chloroplast RNA in Chlamydomonas reinhadtii. RNA. 2000;6(4):598–607. Epub 2000/04/29. 10786850 10.1017/s1355838200992252 PMC1369940

[46] Brian DA, Baric RS. Coronavirus genome structure and replication. Curr Top Microbiol Immunol. 2005;287:1–30. Epub 2004/12/22. . 15609507 10.1007/3-540-26765-4_1 PMC7120446

[47] Brown CG, Nixon KS, Senanayake SD, Brian DA. An RNA stem-loop within the bovine coronavirus nsp1 coding region is a cis-acting element in defective interfering RNA replication. J Virol. 2007;81(14):7716–24. Epub 2007/05/04. JVI.00549-07 [pii] 10.1128/JVI.00549-07 17475638 PMC1933353

[48] Gustin KM, Guan BJ, Dziduszko A, Brian DA. Bovine coronavirus nonstructural protein 1 (p28) is an RNA binding protein that binds terminal genomic cis-replication elements. J Virol. 2009;83(12):6087–97. Epub 2009/04/10. JVI.00160-09 [pii] 10.1128/JVI.00160-09 19357173 PMC2687364

[49] Wu HY, Brian DA. 5'-proximal hot spot for an inducible positive-to-negative-strand template switch by coronavirus RNA-dependent RNA polymerase. J Virol. 2007;81(7):3206–15. Epub 2007/01/19. JVI.01817-06 [pii] 10.1128/JVI.01817-06 17229702 PMC1866079

[50] Jalkanen AL, Coleman SJ, Wilusz J. Determinants and implications of mRNA poly(A) tail size—does this protein make my tail look big? Semin Cell Dev Biol. 2014;34:24–32. Epub 2014/06/10. 10.1016/j.semcdb.2014.05.018 S1084-9521(14)00173-6 [pii]. 24910447 PMC4163081

[51] Ke TY, Liao WY, Wu HY. A Leaderless Genome Identified during Persistent Bovine Coronavirus Infection Is Associated with Attenuation of Gene Expression. PLoS One. 2013;8(12):e82176 Epub 2013/12/19. 10.1371/journal.pone.0082176 PONE-D-13-28779 [pii]. 24349214 PMC3861326

[52] Wu HY, Guy JS, Yoo D, Vlasak R, Urbach E, Brian DA. Common RNA replication signals exist among group 2 coronaviruses: evidence for in vivo recombination between animal and human coronavirus molecules. Virology. 2003;315(1):174–83. Epub 2003/11/01. S0042682203005117 [pii]. . 14592769 10.1016/S0042-6822(03)00511-7 PMC7126556

[53] Brown CE, Sachs AB. Poly(A) tail length control in Saccharomyces cerevisiae occurs by message-specific deadenylation. Mol Cell Biol. 1998;18(11):6548–59. Epub 1998/10/17. 9774670 10.1128/mcb.18.11.6548 PMC109240

[54] Yamashita A, Chang TC, Yamashita Y, Zhu W, Zhong Z, Chen CY, et al Concerted action of poly(A) nucleases and decapping enzyme in mammalian mRNA turnover. Nat Struct Mol Biol. 2005;12(12):1054–63. Epub 2005/11/15. nsmb1016 [pii] 10.1038/nsmb1016 . 16284618

[55] Ahlquist P, Kaesberg P. Determination of the length distribution of poly(A) at the 3' terminus of the virion RNAs of EMC virus, poliovirus, rhinovirus, RAV-61 and CPMV and of mouse globin mRNA. Nucleic Acids Res. 1979;7(5):1195–204. Epub 1979/11/10. 229465 10.1093/nar/7.5.1195 PMC342296

[56] Kempf BJ, Kelly MM, Springer CL, Peersen OB, Barton DJ. Structural features of a picornavirus polymerase involved in the polyadenylation of viral RNA. J Virol. 2013;87(10):5629–44. Epub 2013/03/08. 10.1128/JVI.02590-12 JVI.02590-12 [pii]. 23468507 PMC3648189

[57] Kempf BJ, Barton DJ. Picornavirus RNA polyadenylation by 3D(pol), the viral RNA-dependent RNA polymerase. Virus Res. 2015;206:3–11. Epub 2015/01/07. 10.1016/j.virusres.2014.12.030 S0168-1702(14)00544-9 [pii]. . 25559071 PMC4801031

[58] Silvestri LS, Parilla JM, Morasco BJ, Ogram SA, Flanegan JB. Relationship between poliovirus negative-strand RNA synthesis and the length of the 3' poly(A) tail. Virology. 2006;345(2):509–19. Epub 2005/11/22. S0042-6822(05)00675-6 [pii] 10.1016/j.virol.2005.10.019 . 16297425

[59] Hardy RW, Rice CM. Requirements at the 3' end of the sindbis virus genome for efficient synthesis of minus-strand RNA. J Virol. 2005;79(8):4630–9. Epub 2005/03/30. 79/8/4630 [pii] 10.1128/JVI.79.8.4630-4639.2005 15795249 PMC1069581

[60] Liu P, Yang D, Carter K, Masud F, Leibowitz JL. Functional analysis of the stem loop S3 and S4 structures in the coronavirus 3'UTR. Virology. 2013;443(1):40–7. Epub 2013/05/21. 10.1016/j.virol.2013.04.021 S0042-6822(13)00230-4 [pii]. 23683838 PMC3700632

[61] Zust R, Miller TB, Goebel SJ, Thiel V, Masters PS. Genetic interactions between an essential 3' cis-acting RNA pseudoknot, replicase gene products, and the extreme 3' end of the mouse coronavirus genome. J Virol. 2008;82(3):1214–28. Epub 2007/11/23. JVI.01690-07 [pii] 10.1128/JVI.01690-07 18032506 PMC2224451

[62] Ivshina M, Lasko P, Richter JD. Cytoplasmic polyadenylation element binding proteins in development, health, and disease. Annu Rev Cell Dev Biol. 2014;30:393–415. Epub 2014/07/30. 10.1146/annurev-cellbio-101011-155831 . 25068488

[63] Burd CG, Matunis EL, Dreyfuss G. The multiple RNA-binding domains of the mRNA poly(A)-binding protein have different RNA-binding activities. Mol Cell Biol. 1991;11(7):3419–24. Epub 1991/07/01. 1675426 10.1128/mcb.11.7.3419-3424.1991 PMC361068

[64] Kuhn U, Pieler T. Xenopus poly(A) binding protein: functional domains in RNA binding and protein-protein interaction. J Mol Biol. 1996;256(1):20–30. Epub 1996/02/16. S0022-2836(96)90065-0 [pii] 10.1006/jmbi.1996.0065 . 8609610

