# Supplementary figures and images for: Characterization of the Role of Hexamer AGUAAA and Poly(A) Tail in Coronavirus Polyadenylation
Source: PLoS One. 2016 Oct 19;11(10):e0165077. doi: 10.1371/journal.pone.0165077 (PMC5070815; doi:10.1371/journal.pone.0165077)

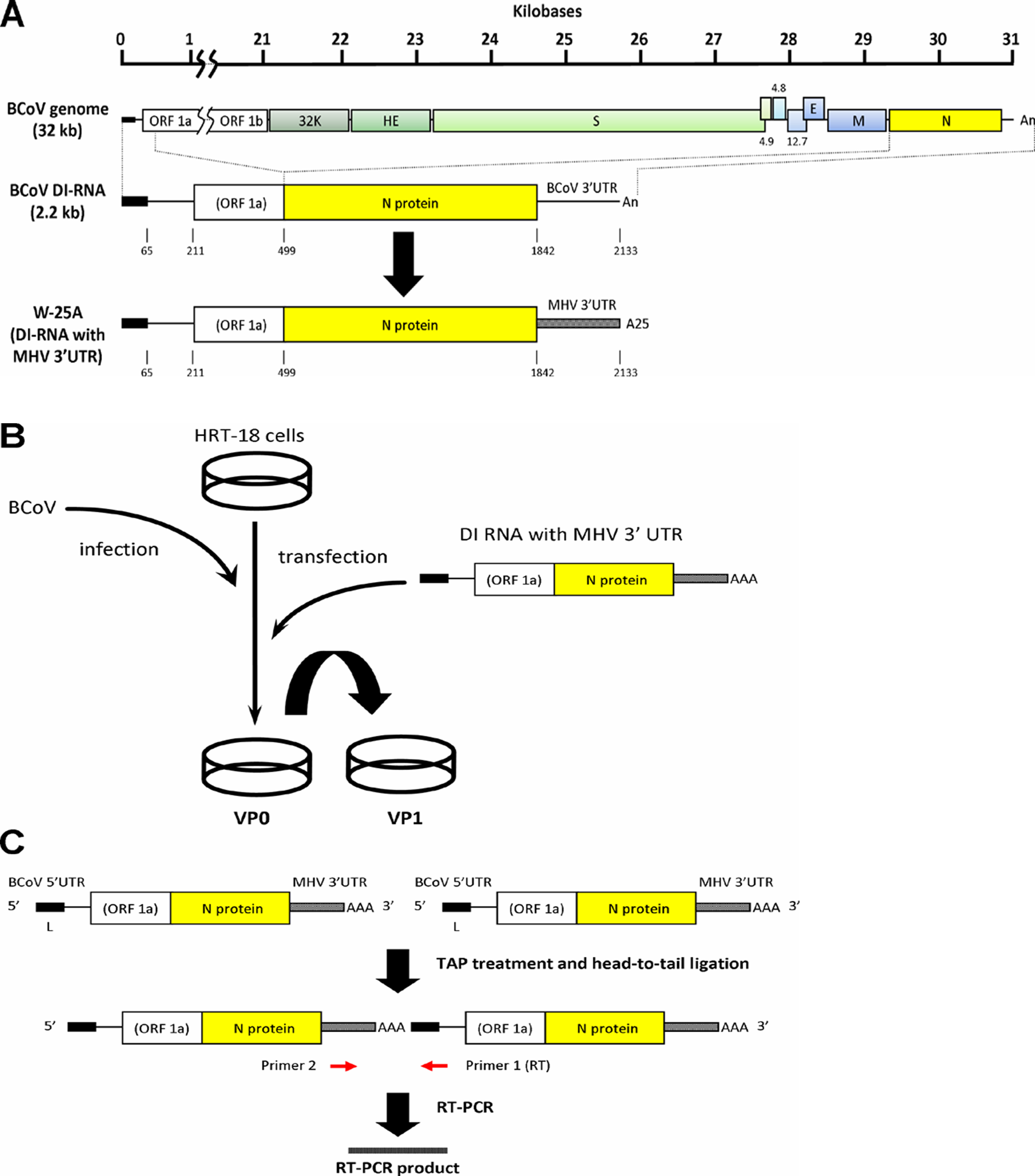

Supplement: S1 Fig — (A) The structure of BCoV DI RNA and its composition relative to the BCoV genome are illustrated in the upper panel. The engineered BCoV DI RNA with the MHV 3’ UTR (W-25A) used in this study is shown in the lower panel. To differentiate the origin of the poly(A) tail between the helper virus BCoV genome and BCoV DI RNA, the latter was engineered to carry the mouse hepatitis virus (MHV) 3’ UTR with which an MHV-specific primer can be used for RT-PCR to determine the length of the DI RNA poly(A) tail. (B) Schematic illustration of the experiment showing the transfection of BCoV-infected HRT-18 cells with DI RNA and the passage of the resulting BCoV progeny. (C) Method for determining the coronaviral poly(A) tail length. The decapped and head-to-tail ligated BCoV DI RNA was used as the template for RT-PCR with primer 1 (for RT) and primer 2 followed by sequencing. (TIF) [file pone.0165077.s001.tif]

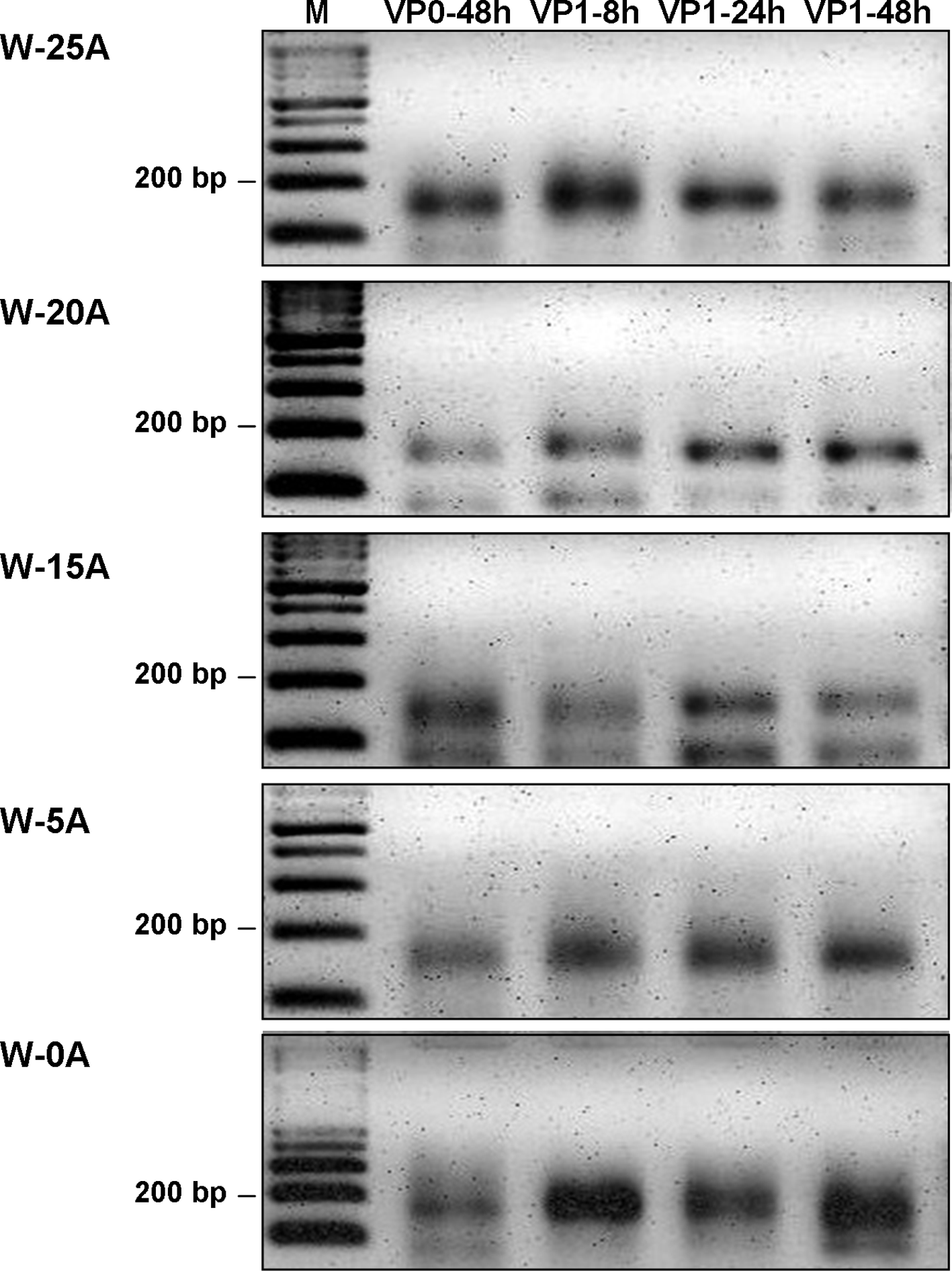

Supplement: S2 Fig — (TIF) [file pone.0165077.s002.tif]

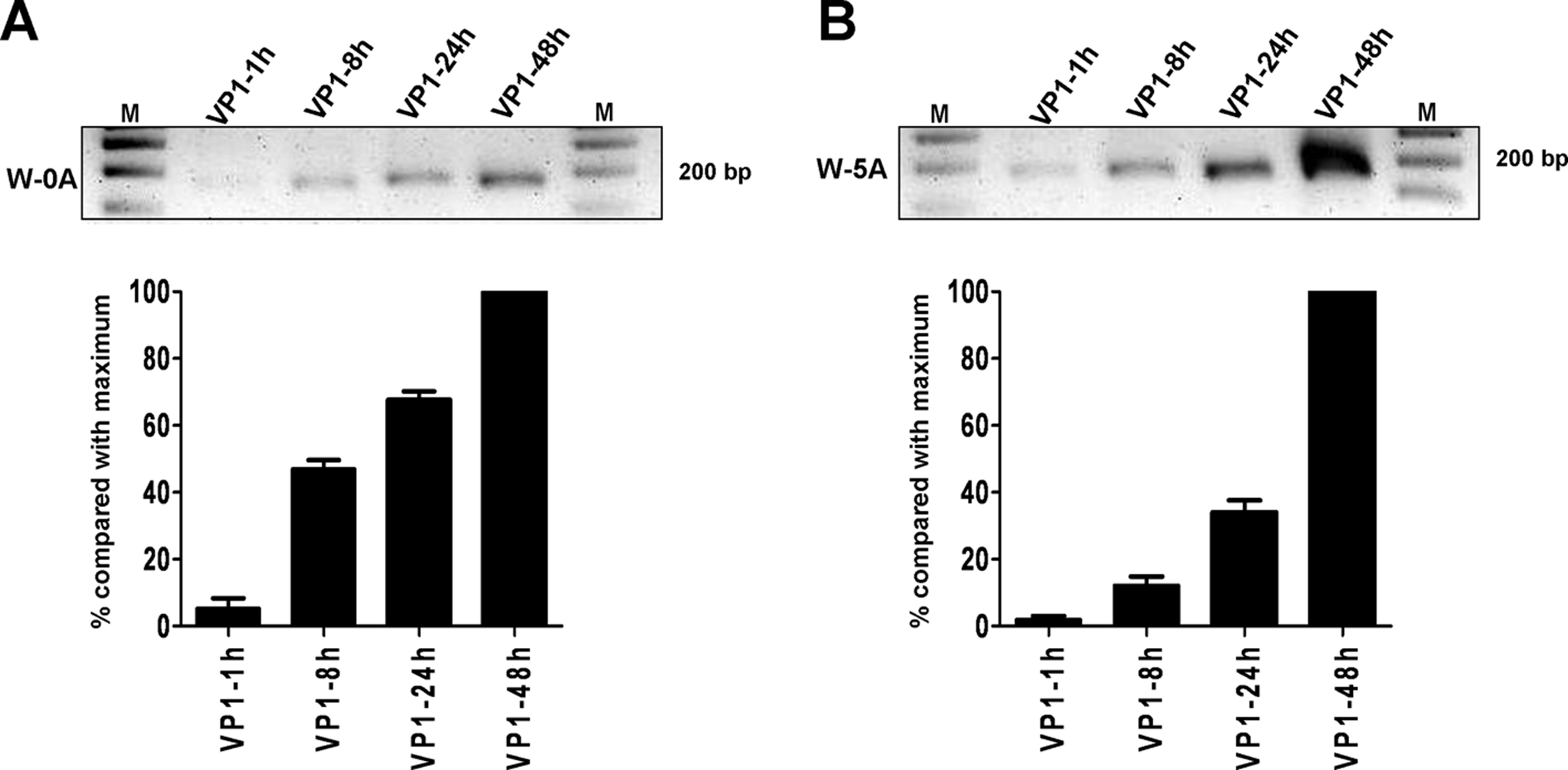

Supplement: S3 Fig — (A) and (B) Upper panel: detection of DI RNAs W-0A and W-5A at the different time points of VP1 by RT-PCR. (A) and (B) Lower panel: quantitation of the synthesis of DI RNAs W-0A and W-5A at the different time points of VP1 by qRT-PCR. (TIF) [file pone.0165077.s003.tif]

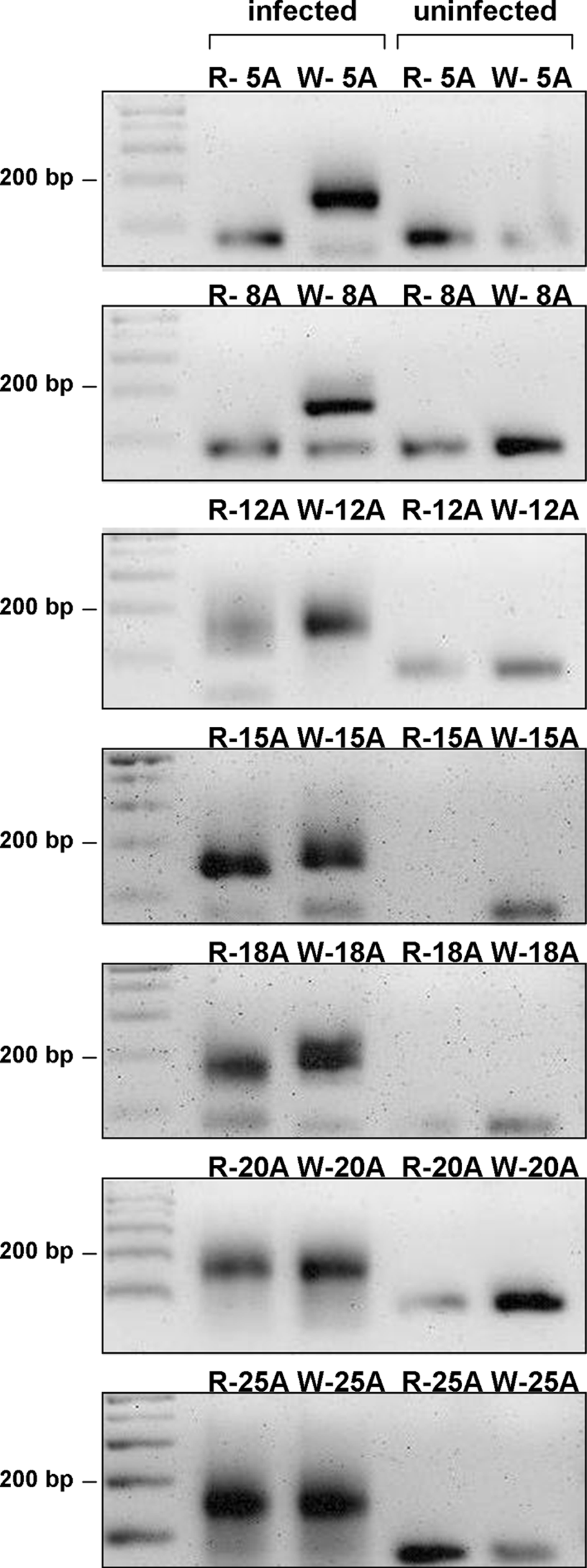

Supplement: S4 Fig — (TIF) [file pone.0165077.s004.tif]

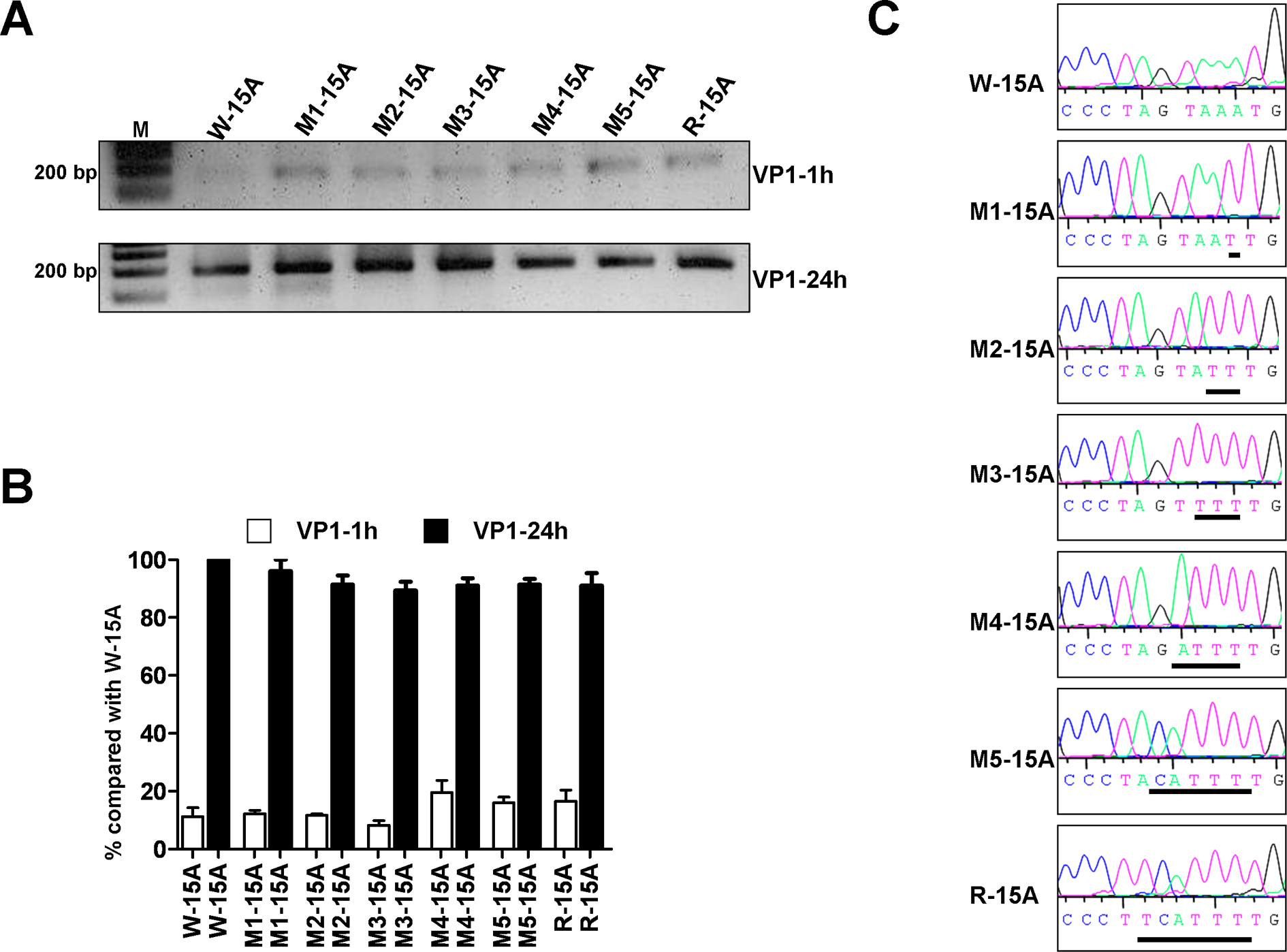

Supplement: S5 Fig — (A) Detection of DI RNA synthesis at 1 and 24 hpi of VP1 by RT-PCR. (B) The relative levels of DI RNA synthesis at 1 and 24 hpi of VP1 as quantitated by qRT-PCR. (C) Sequence analysis of DI RNA at 24 hpi of VP1. The sequence within the hexamer AGUAAA of individual DI RNA different from that of DI RNA W-15A is underlined. (TIF) [file pone.0165077.s005.tif]

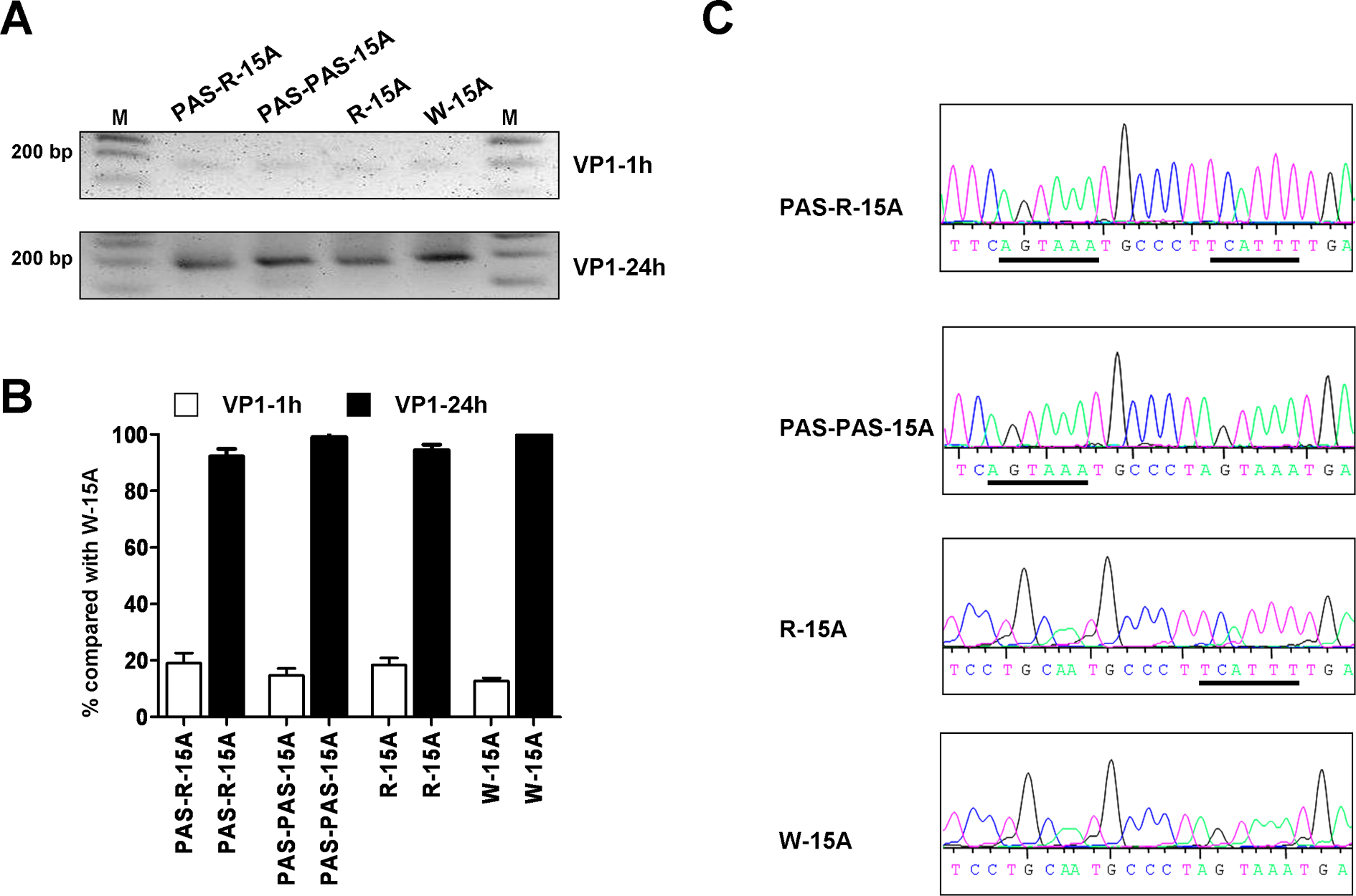

Supplement: S6 Fig — (A) Detection of DI RNA synthesis at 1 and 24 hpi of VP1 by RT-PCR. (B) The relative levels of DI RNA synthesis at 1 and 24 hpi of VP1 as quantitated by qRT-PCR. (C) Sequence analysis of DI RNA at 24 hpi of VP1. The sequence of individual DI RNA different from that of DI RNA W-15A is underlined. (TIF) [file pone.0165077.s006.tif]

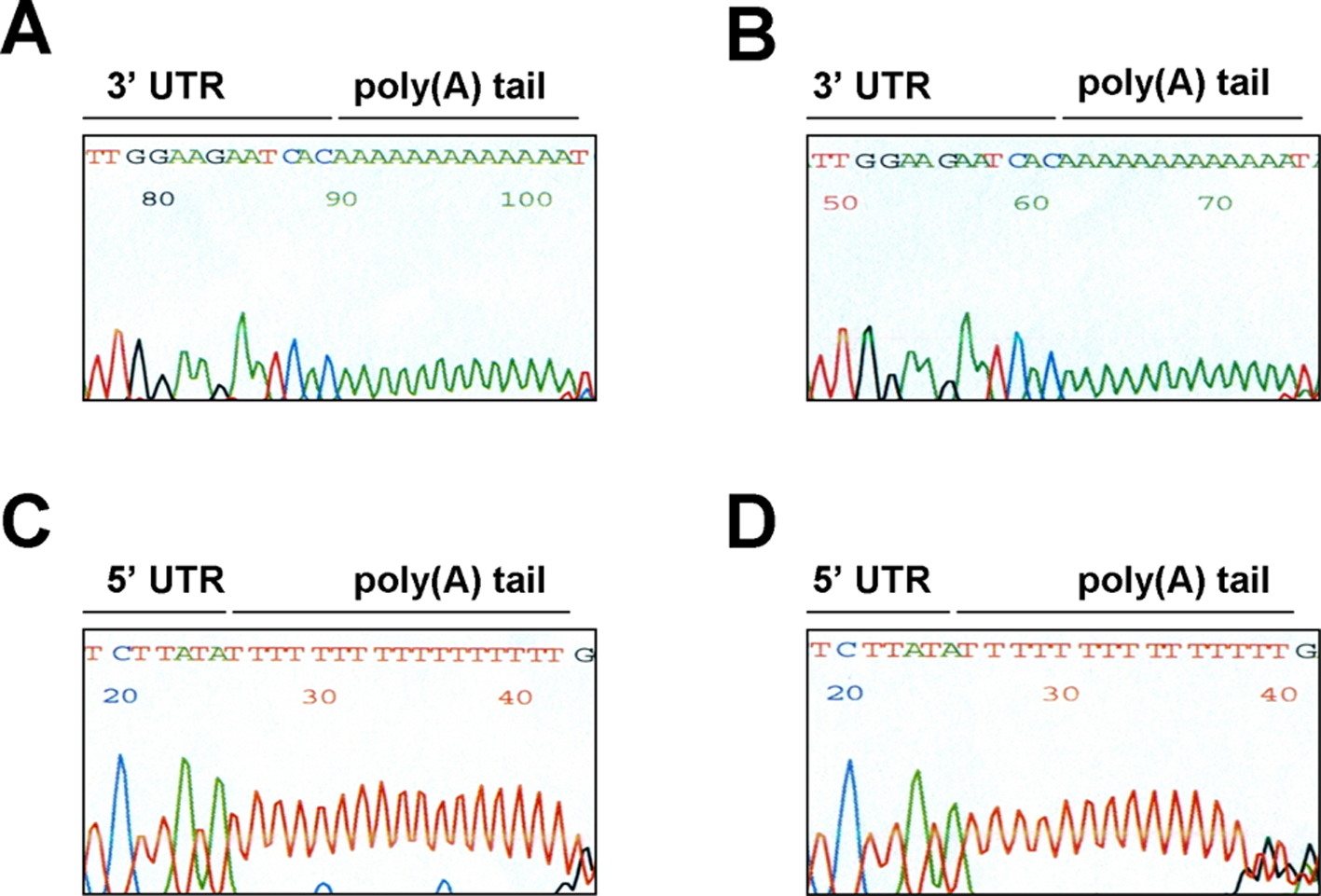

Supplement: S7 Fig — (A) BCoV-infected HRT-18 cells at 2 hpi (13 nts). (B) W-25A-transfected BCoV-infected HRT-18 cells at 72 hpi of VP1 (13 nts). (C) Mouse brain at 5 days postinfection with MHV-A59 (17 nts) (shown in negative strand). (D) Delayed brain tumor (DBT) cells with MHV persistent infection of 97 days (15 nts) (shown in negative strand). (TIF) [file pone.0165077.s007.tif]

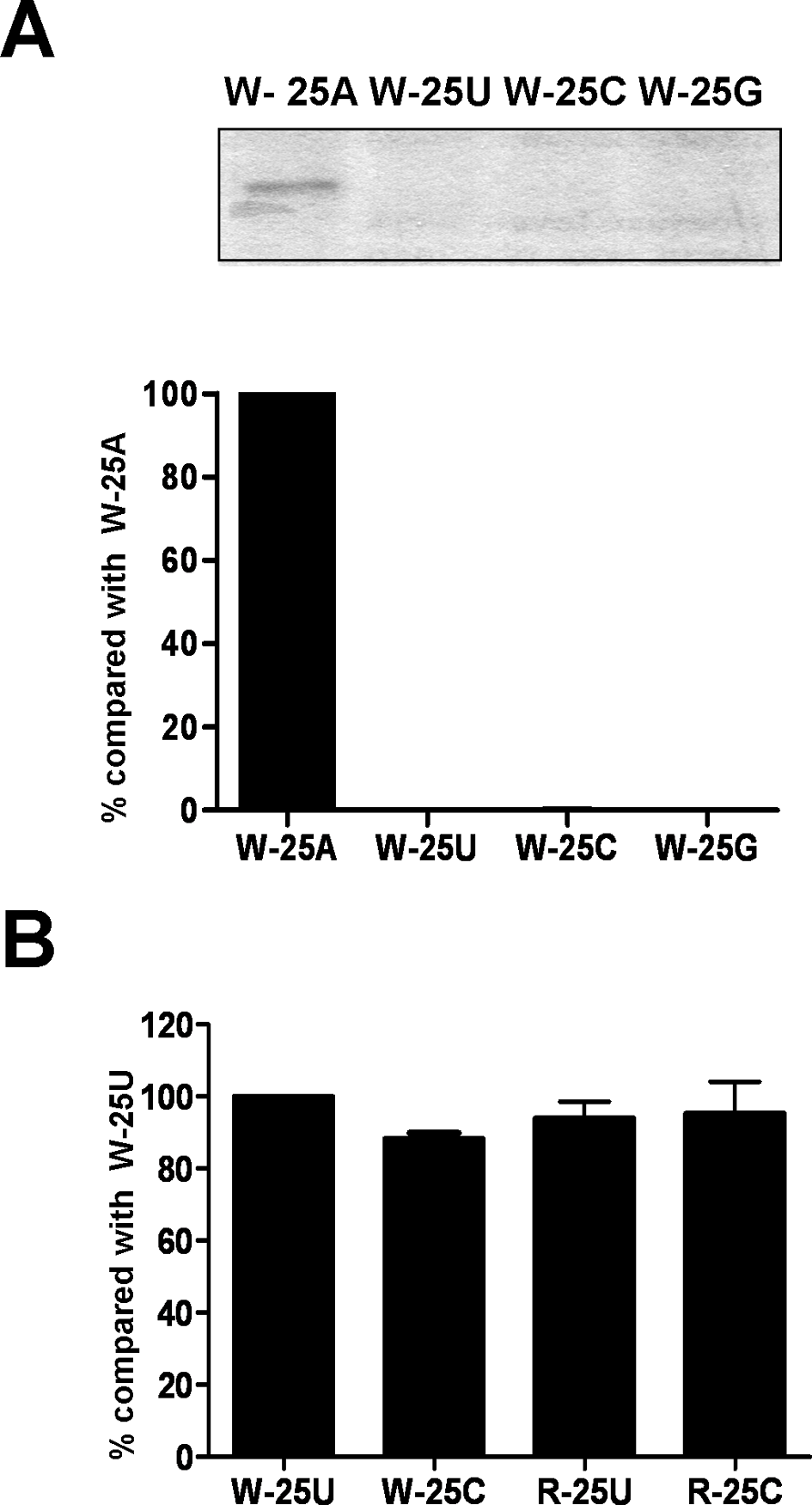

Supplement: S8 Fig — (A) Upper panel: synthesis of W-25A, W-25U, W-25C and W-25G at 48 hpi of VP1 as detected by Northern blot analysis. Lower panel: the relative levels of RNA synthesis. (B) Quantitation of the synthesis of DI RNAs W-25U, W-25C, R-25U and R-25C at 24 hpi of VP1 by qRT-PCR. (TIF) [file pone.0165077.s008.tif]

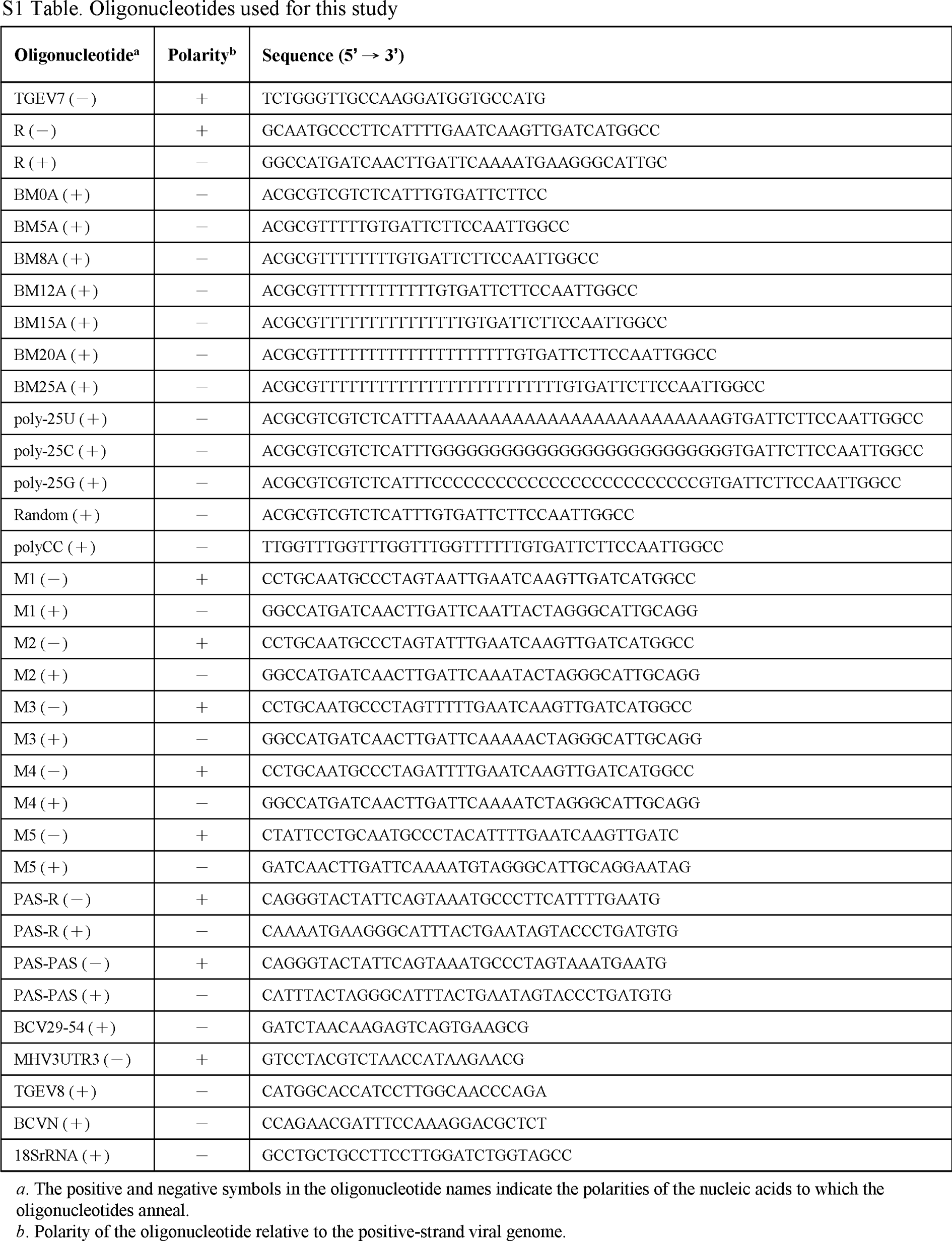

Supplement: S1 Table — (TIF) [file pone.0165077.s009.tif]
